# Supplementary material for: Symmetry-breaking in patch formation on triangular gold nanoparticles by asymmetric polymer grafting
Source: Nat Commun. 2022 Nov 9;13:6774. doi: 10.1038/s41467-022-34246-0 (PMC9646788; doi:10.1038/s41467-022-34246-0)
Supplement: Supplementary file 1 — Supplementary Information [file 41467_2022_34246_MOESM1_ESM.pdf]

Supplementary Information

Symmetry-breaking in patch formation on triangular gold nanoparticles by asymmetric polymer grafting

Ahyoung Kim,[†] Thi Vo,[†] Hyosung An, Progna Banerjee, Lehan Yao, Shan Zhou, Chansong Kim, Delia J. Milliron, Sharon C. Glotzer,[*] Qian Chen[*]

[†] These authors contributed equally to this work.
[*] Correspondence to: sglotzer@umich.edu, qchen20@illinois.edu

Contents

Supplementary Notes 1–9

Supplementary Tables 1–6

Supplementary Figures 1–21

Supplementary References

Supplementary Notes 1–9

Supplementary Note 1: χ dependent excluded volume and chain scaling behaviors of polystyrene-*b*-polyacrylic acid (PS-*b*-PAA) adsorbed onto nanoprisms

Previous theories[1,2] developed for chain partitioning on the surface of anisotropic nanoparticles (NPs) explained the driving force localizing chains to the vertices of anisotropic NPs, using athermal systems without chain-chain interactions. Briefly, locations of higher curvature on the surface of anisotropic NPs afford more volume per solid angle with increasing radial distance away from the NP surface as in Fig. 2a. Thus, polymers anchored to NPs surface of equivalently high curvature have similarly less entropic cost of grafting, leading to symmetric grafting. However, such energetic cost only, cannot explain why symmetry-breaking occurs regardless of similar local curvature on the nanoprism vertices.

To incorporate intermolecular enthalpy into PS-*b*-PAA in solvent system, we made two assumptions. PS-PS interaction dominates over that of PAA-PAA and PAA-blocks minimizes their contact with PS-blocks and faces the solvent in equilibrium. These assumptions are based on the fact that the volume fraction of PS-blocks is 0.86 and their interaction with solvent molecules is unfavorable compared to that of PAA, consistent with established observations that highly asymmetry (low PS-block fraction) block polymers form micelles in solution[3]. Making the above assumptions allow us to split the polymeric grafts into two layers – internal PS-block and external PAA-block. The outer PAA-block governs patch-solvent stability and the internal PS-block drives chain partitioning.

We additionally note that DMF—a good solvent for PS—constitutes the 82% of the solvent composition. This suggests that perturbations about athermal grafting theories can provide a good first order approximation[1,2,4]. Taking a page from developed theories for planar brushes, we choose to account for deviations away from good solvent behaviors *via* modifying the Flory interaction parameter χ of PS-solvent[5,6]. More specifically, since the presence of water decreases the solubility of the PS-block, we work in the limit of $\chi > 0$. From a scaling perspective, the effect of nonzero χ is incorporated into the excluded volume ν such that $\nu \sim \nu_{\mathrm{o}}(1-2\chi)$, where ν_{o} is the unperturbed good solvent excluded volume. While this relation is well-known in the polymer physics community, we provided its derivation here to better help with clarity. Our starting point is the traditional polymer extension to regular solution theory, where the mean free energy of mixing $\overline{F_{\mathrm{m}}}$ can be written as

$$\beta\overline{F_{\mathrm{m}}} = \frac{\phi_{\mathrm{P}}}{N}\ln\phi_{\mathrm{P}} + \frac{1-\phi_{\mathrm{P}}}{N_{\mathrm{S}}}\ln(1-\phi_{\mathrm{P}}) + \chi\phi_{\mathrm{P}}(1-\phi_{\mathrm{P}}) \tag{1}$$

where ϕ_{P} is the polymer volume fraction, N is the polymer chain length, N_{S} is the solvent chain length (typically $N_{\mathrm{S}} = 1$, but we keep it as a variable for generalizability), and $\beta = 1/k_{\mathrm{B}}T$. Taylor expansion of $\ln(1-\phi_{\mathrm{P}})$ in the $\phi_{\mathrm{P}} \ll 1$ limit gives

$$\beta\overline{F_{\mathrm{m}}} = \frac{\phi_{\mathrm{P}}}{N}\ln\phi_{\mathrm{P}} + \phi_{\mathrm{P}}\left(\chi - \frac{1}{N_{\mathrm{S}}}\right) + \frac{1}{2}\phi_{\mathrm{P}}^2\left(\frac{1}{N_{\mathrm{S}}} - 2\chi\right) + \frac{1}{6}\frac{\phi_{\mathrm{P}}^3}{N_{\mathrm{S}}} + O\left(\phi_{\mathrm{P}}^4\right) \tag{2}$$

We then define the osmotic pressure Π such that $\Pi = -\partial\overline{F_{\mathrm{m}}}/\partial V$, with $V = b_l^3 n_{\mathrm{P}} N/\phi$ where b_l is the Kuhn length of the polymer of interest and n_{P} is the number of chains within the system. The relation for V is simply the definition of the volume fraction. Applying the derivative and accounting for change of variables gives (dropping terms of order ϕ_{P}^4 and higher):

$$\beta\Pi = b_l^{-3}\left[\frac{\phi_{\mathrm{P}}}{N} + \frac{1}{2}\phi_{\mathrm{P}}^2\left(\frac{1}{N_{\mathrm{S}}} - 2\chi\right) + \frac{1}{3}\frac{\phi_{\mathrm{P}}^3}{N_{\mathrm{S}}}\right] \tag{3}$$

We then note that $\phi_{\mathrm{P}} b_l^{-3}$ is simply the polymer number density η_{P}, substituting into Eq. 3

$$\beta\Pi = \frac{\eta_P}{N} + \frac{1}{2}\left[b_l^3\left(\frac{1}{N_S} - 2\chi\right)\right]\eta_P^2 + \frac{1}{3}\frac{b_l^6}{N_S}\eta_P^3 \tag{4}$$

Eq. 4 is the virial expansion in density: $\beta\Pi = B_1\eta_p + 2^{-1}B_2\eta_p^2 + \cdots = \sum_{i=1}^{\infty} i^{-1}B_i\eta_P^i$. As such, the excluded volume is the second virial coefficient $B_2 = b_l^3(1/N_S - 2\chi)$. b_l^3 is set to be a reference, unperturbed excluded volume $\nu_o \sim b_l^3$. Setting $N_S = 1$ for typical solvents gives $\nu \sim \nu_o(1 - 2\chi)$.

Similar to our works for grafted chain behaviors[7], we define a swelling parameter κ for chains in the Θ ("theta") and good solvent limit.

$$\kappa \sim \frac{R_{\text{good}}}{R_\Theta} \sim \frac{\nu^{1/5}b_l^{2/5}N^{3/5}}{N^{1/2}b_l} \sim \nu^{1/5}b_l^{-3/5}N^{1/10} \tag{5}$$

Plugging in $\nu \sim \nu_o(1 - 2\chi)$ and defining a dimensionless volume $v_o \sim \nu_o b_l^{-3}$ gives

$$\kappa^5 \sim v_o[1 - 2\chi]N^{1/2} \tag{6}$$

Eq. 6, however, operates in the limit of free chains in solution. Extending to grafting on the surface of an anisotropic particle (such as triangular prisms), we employ four geometrical scaling balances. This set of derivations is analogous to that for linear and branching chains with $\chi = 0$ previously developed by our group[1,7,8].

Geometric Balance 1: Local monomer density = global monomer density

$$\psi(r) \sim \frac{n(r)b^3}{\xi^3(r)} \sim \frac{n(r)fb_l^3}{r^2\Omega^3\xi(r)} \tag{7}$$

Geometric Balance 2: Correlation size swells relative to Θ-solvent behavior

$$\xi(r) \sim \kappa(r)\xi_o(r) \tag{8}$$

Geometric Balance 3: Unswollen correlation size = Θ-solvent chain size

$$\xi_o(r) \sim n^{1/2}(r)b_l \tag{9}$$

Geometric Balance 4: Chain swelling (Eq. 6)

$$\kappa^5(r) \sim v_o[1 - 2\chi]n^{1/2}(r) \tag{10}$$

where ξ is the correlation blob size, above which the intramolecular interaction vanishes due to the presence of other polymer chains, n is the monomer count at distance r from the core's surface, ψ is the monomer volume fraction, Ω is a core shape parameter, and f is the number of grafts. f is related to the grafting density σ via $\sigma \sim f/r_o^2$ with r_o representing the nanoprism in-sphere radius. Physically geometric balances 1 – 4 (Eq. 7 – 10) define volume filling constraints for all grafted chains. More specifically, they make the critical assumption that chains will swell/shrink to fill all possible space available. Relative degrees of swelling are quantified relative to the gaussian, Θ-solvent limit since this is the chain's ideal

state. Enforcing equality between local and global density ensures that the chemical potential is the same at all radially equivalent positions relative to a particle's surface. In other words, we assume that chains are free to reorganize and seek to predict their equilibrium behavior. Solving the coupled set of equations gives

$$\xi(r) \sim r f^{-1/2} \Omega^{3/2} \tag{11}$$

$$n(r) \sim \left(\frac{r}{b_l}\right)^{5/3} \Omega^{5/2} f^{-5/6} v_o^{-1/3} [1 - 2\chi]^{-1/3} \tag{12}$$

$$\kappa(r) \sim \left(\frac{r}{b_l}\right)^{1/6} \Omega^{1/4} f^{-1/12} v_o^{1/6} [1 - 2\chi]^{1/6} \tag{13}$$

$$\psi(r) \sim \left(\frac{r}{b_l}\right)^{-4/3} \Omega^{-2} f^{2/3} v_o^{-1/3} [1 - 2\chi]^{-1/3} \tag{14}$$

Eq. 11 – 14 operate in the regime where solvent quality plays a role in controlling the amount of swelling and number of monomers present in each correlation blob. Geometry sets the correlation blob size at a given distance from the core (Eq. 11). Solvent quality—$v_o[1 - 2\chi]$—dictates how many monomers fill the given correlation blob ξ (Eq. 12), which then controls both the relative degree of swelling (Eq. 13) and monomer density (Eq. 14).

Moving closer to the core, there exists a crossover distance where the monomer density becomes so high that no solvent can penetrate the grafted layer. This means that monomers sitting within this region effectively sees only monomers from either the same or neighboring chains. In essence, monomers within this regime will behave as if they are sitting in a melt and has no driving force to swell. Setting the swelling parameter $\kappa(r) \sim 1$, defines this crossover distance r_θ to be

$$r_\theta \sim b_l \Omega^{-3/2} f^{1/2} v_o^{-1} [1 - 2\chi]^{-1} \tag{15}$$

The corresponding relations for $n(r)$, $\xi(r)$, and $\psi(r)$ are then

$$\xi(r) \sim r f^{-1/2} \Omega^{3/2} \tag{16}$$

$$n(r) \sim \left(\frac{r}{b_l}\right)^{2} \Omega^{3} f^{-1} \tag{17}$$

$$\psi(r) \sim \left(\frac{r}{b_l}\right)^{-1} \Omega^{-3/2} f^{1/2} \tag{18}$$

Similar to Eq. 11, the correlation size (Eq. 16) is defined purely based on geometry and is therefore identical. However, since no solvent can penetrate the region where $r < r_\theta$, the effect of solvent interactions (χ-dependence) necessary disappear in Eq. 17 – 18.

To be rigorous, there exists an additional regime where there is high crowding between monomers that chains are forced to fully stretch. Here, the monomer density becomes uniform. Setting $\psi(r) \sim 1$ gives

$$r_{\mathrm{c}} \sim b_l \Omega^{-3/2} f^{1/2} \tag{19}$$

Trivially, $n(r) \sim 1$, $\kappa(r) \sim 1$, and $\xi(r) \sim b_l$ within this regime ($r < r_{\mathrm{c}}$). To obtain the chain size, we integrate $\psi(r)$ across all regimes using the following volumetric balance ($d\vec{r} \sim r^2\Omega^3 dr$)

$$Nfb_l^3 \sim \int_0^R \psi(r) d\vec{r} \tag{20}$$

Expanding over the relevant regimes gives

$$\begin{aligned} Nfb_l^3 \sim & \int_0^{r_{\mathrm{c}}} r^2\Omega^3 dr + \int_{r_{\mathrm{c}}}^{r_\theta} b_l f^{1/2} r \Omega^{3/2} dr \\ & + \int_{r_\theta}^{R} b_l^{4/3} f^{2/3} v_{\mathrm{o}}^{-1/3} [1-2\chi]^{-1/3} r^{2/3} \Omega dr \end{aligned} \tag{21}$$

Integrating and keeping the relevant terms with χ dependency gives the end-to-end distance R for a polymer chain anchored to different locations on the nanoprism surface

$$R \sim r_{\mathrm{o}} \sigma^{1/5} v_{\mathrm{o}}^{1/5} [1-2\chi]^{1/5} b_l^{2/5} \left[\frac{N}{\Omega}\frac{b_l}{r_{\mathrm{o}}}\right]^{3/5} \left\{1 - r_{\mathrm{o}}\sigma^{1/2}\Omega^{-3/3} v_{\mathrm{o}}^{-2} \frac{2\chi[1-\chi]}{[1-2\chi]^2}\frac{1}{N}\right\} \tag{22}$$

In the limit of long chains ($N \gg 1$) Eq. 22 simplifies to

$$R \sim r_{\mathrm{o}} \sigma^{1/5} v_{\mathrm{o}}^{1/5} [1-2\chi]^{1/5} b_l^{2/5} \left[\frac{N}{\Omega}\frac{b_l}{r_{\mathrm{o}}}\right]^{3/5} \tag{23}$$

By inspection, Eq. 23 is simply the results for grafted chains for $\chi = 0$ (Eq. 24) multiplied by the additional factor of $[1-2\chi]^{1/5}$.

$$R \sim r_{\mathrm{o}} \sigma^{1/5} v^{1/5} b_l^{2/5} \left[\frac{N}{\Omega}\frac{b_l}{r_{\mathrm{o}}}\right]^{3/5} \tag{24}$$

In our limit of interest, where $\chi > 0$, Eq. 18 is such that the chain shrinks since $[1-2\chi]^{1/5} < 1$. Physically, this captures the phenomenon that the chains slightly prefer themselves over the solvent. The grafted layer partially shrinks to enhance monomer-monomer interactions. In effect, Eq. 18 performs a perturbation about Eq. 24 where the size of the grafted layer either expands or contracts to account for changes between solvent–chain and chain–chain interactions.

Here, one could argue heuristically that Eq. 23 can be derived by replacing v with $v_{\mathrm{o}}[1-2\chi]$ in Eq. 24. While this does capture the physical picture of perturbations in solvent quality, deriving Eq. 11 – 23 enables us to derive a cross-over χ value where chain-chain interactions become strong enough to induce the symmetry breaking in patch formation on NPs. This is of particular interest in guiding experimental selection of solvent/grafting conditions.

Our logical scaling argument is as follows. With small to zero changes in solvent quality, the driving force favoring chain-chain interactions is small and the system should still exhibit good solvent chain scaling as derived in Eq. 22 (or Eq. 23). Conversely, in the high reduction in solvent quality limit, solvent molecules are squeezed out of the grafted chain layer in favor of more chain-chain interaction. If χ becomes 0.5, v is zero and create a Θ-like environment for polymer chains in solution. This results in a majority of

the grafted chain layer transitioning to exhibit a "melt-like" state as only chain-chain interactions are present. The transition from solvent wetting to melt-like directly mirrors the emergence of symmetry breaking patches we seek to take advantage of in experiments. We derive the "melt-like" chain size using Eq. 25

$$Nfb_l^3 \sim \int_0^{r_c} r^2\Omega^3 dr + \int_{r_c}^{R_\Theta} b_l f^{1/2} r\Omega^{3/2} dr \tag{25}$$

Taking the limit of $N \gg 1$, we solve Eq. 24 for the end-to-end chain distance at Θ-like environment R_Θ to give $R_\Theta \sim r_o\sigma^{1/4}b_l^{1/2}N^{1/2}\Omega^{-3/4}(b/r_o)^{1/2}$. Equating R_Θ with Eq. 22 and solving for χ gives the crossover χ^*

$$\chi^* \sim 2^{-1}\left[1 - r_o^{1/2}\sigma^{1/4}\Omega^{-3/4}v_o^{-1}N^{-1/2}\right] \tag{26}$$

Supplementary Note 2: End-attached polymer chain model validation

The polymers attached on the prism surface can be modeled using the end-attached configuration. The major experimental evidence of the polymer chains adopting an end-attached configuration as the patches on the NP surface is the patch thickness (t_m), which is up to 18.9 ± 4.0 nm (Fig. 1e) across the conditions we have sampled in our work. This thickness matches with the expected extended chain length based on the polymers' molecular weight (~22.9 nm, in good solvent). Furthermore, we note that the solvent quality and the PAA block fraction ϕ_A used in our polymer is within the micellar formation regime (i.e., low ϕ_A regime on the block co-polymer phase diagram)[9,10]. On surfaces, this will result in the equilibrium conformation of a double layer—PS inner layer and PAA outer layer. The chains are mobile on the particle surface (see Supplementary Note 4) and thus we assume that they can reorganize into the "end attachment" double layer motif shown in the schematic of Fig. 2a. Since our theory is equilibrium by design, this assumption of the final "end attachment" conformation becomes appropriate for our geometrical scaling balances.

It is also true that when the polymer concentration on the prism surface (i.e., grafting density) is low, several PS units could lie flat on the NP surface, as shown in the NPs collected at 10% reaction procession (Supplementary Fig. 5a). In those cases, the patch thickness is smaller (t_m = 5.8 ± 2.3 nm) due to the more flattened polymer configuration. However, as the system is in equilibrium, PS-*b*-PAA polymers form a double layer with their PS end attached on the prism surface. This configuration in polymer scaling theory still holds the polymer chains to be effectively attached by one end (PS), although the "end" becomes larger as the number of attached PS units is increased.

Supplementary Note 3: Monte Carlo grafting simulation and collapsing into phase diagram

We develop a Monte Carlo grafting algorithm to chain-chain interactions driving the symmetry breaking during the grafting process. Using the chain size R, the free energy of each chain is

$$\beta F \sim \frac{R^2}{Nb_l^2} + \frac{v_o N^2 f}{(\Omega R)^3} \tag{27}$$

Eq. 27 is then employed to compute a Boltzmann weight probability of chain grafting at a given nanoparticle (NP) surface position (defined in Ω) such that $P_{\text{graft}} \sim e^{-\beta F}$, which is then used as the acceptance probability in the standard Metropolis criterion. To graft chains to an anisotropic particle of interest, we start by constructing a mesh of points occupying the particle surface. For each surface point, Ω

is defined such that $\Omega = \|\vec{r}\| \left\{\min_S \|\vec{r_s}\|\right\}^{-1}$. Here, $\vec{r}$ is the vector pointing from the point of interest to the center-of-mass of the surface mesh and $\min_S \|\vec{r_s}\|$ operates over all points on the mesh. To capture chain-chain interaction, a occupancy matrix $\boldsymbol{M}$ is employed to keep track of which surface points is occupied and updated with every new graft addition. All surface points whose distance is within $\xi(r)$ of occupied points as defined in $\boldsymbol{M}$ feels the effect of chain-chain interaction (i.e., $\chi \neq 0$). Specifically, for graft points within $\xi(r)$ of an occupied point Eq. 23 is used for R in Eq. 27. For all points outside this cutoff distance, Eq. 24 is plugged in for R in Eq. 27. This process repeats until the number of grafted chains matches that defined by a targeted grafting density σ.

The crossover phase boundary in Fig. 2j is derived by defining the condition when the enthalpic gain for adding another chain to the growing patch is on the order of thermal noise (i.e. unity). At this transition, chains are stretched outward due to an already high degree of confinement. As such, $R \sim \sigma^{1/3} N^{1/3}$. The scaling balance takes the form $\chi N^2 R^{-3} \sim 1$. From earlier, $\chi \sim \sigma^{1/4} N^{-1/2} T^{-1}$. Plugging in χ and rearranging terms give $T \sim \sigma^{-3/4} N^{1/2}$. We employed this relationship to draw the theoretical phase boundary as well as collapsing 3D data onto a 2D phase diagram in Fig. 2j.

Supplementary Note 4: Interaction range of polymer chains on the prism tip

We note that the distance between two neighboring tips (62.5 nm) is greater than the coiled chain size of 9.3 nm (assuming good solvent). However, there are two things worth noting here. Firstly, the size of a chain decorating the tip of the prism is generally more extended than that of a free coil. A higher extension implies that the interaction range between a free chain and chain decorating the tip effective increases. Secondly, chains can reorganize. Therefore, stochastically adsorbed chains on a second tip could detach into the solution or move around the particle surface to find the most energetically favorable sites. In other words, as long as the maximal extension length of one chain falls within the maximal range of the other, the chains can reorganize to an *equilibrium* single patch formation, as confirmed via experimental observations of high-yield single-patch prisms (Fig. 1) and simulations of the polymer adsorption process (Fig. 2h–i).

To help with clarity, we plot the monomer density distribution, $\phi(r)$ (predicted from theory), as a function of distance from the tip (Supplementary Fig. 9). One chain is placed at a reference tip ($r = 0$) and the other at a distance equal to the edge length of the prism (R_{tip}). Single dashed lines mark the *equilibrium*, hydrodynamic size of the tip-decorating chain (R_{chain}) and double dashed lines indicate the fully extended size of the chain (R_{rigid}). Colors correspond to the same chain (i.e., blue: chain at reference tip, orange: chain at adjacent tip) and the x-axis is scaled by R_{chain}. The gray shading region indicates the region of space where the chains monomer densities overlap with each other. This means that any monomer within this region will experience strong monomer–monomer interactions that ultimately drive chain reorganization and subsequent merging to a single tip.

Supplementary Note 5: Quantitative Analysis of the TEM images

The projected area of a polymer patch (Fig. 3c) was obtained from TEM images using built-in “Binary” function of ImageJ with low intensity thresholds to segment polymer patches, assisted by manual correction of the patch contour. The local thickness of a polymer patch (Fig. 3a) is defined as the diameter of the largest circle that incorporates the outmost pixel of the patch boundaries and remain within the polymer patch. We obtained local thickness using the built-in function “local thickness” on binary images in open-source software ImageJ, as detailed in our previous work[11,12].

The local curvature at each coordinate of the contours of the prism (Supplementary Fig. 4) and patch (Figs. 3e, 4c, Supplementary Fig. 14g, and 16f) is defined as the inverse of the radius of the best-fitted circle

to the local arc at the coordinate. The contour coordinates of prism and polymer patches are obtained from the segmented images using the MATLAB built-in function “bwboundaries.m”. The coordinates are then plugged into our customized MATLAB code to calculate curvatures with optimized parameters (e.g, 12 pixels, 5.0 nm; fitted by 25 pixels, 10.3 nm), as detailed in our previous work[12]. For the local curvature of the merged patches (Fig. 4c, Supplementary Fig. 16f), we use the multi-point function in ImageJ to manually annotate the contour of the merged patches between a pair of single-patch nanoprisms. The coordinates of the annotated contours are saved in .csv files and then output into the customized MATLAB code to compute the local curvature values as described above.

The patch width $w(x)$ (Fig. 3f) was measured based on segmented images using our customized codes written in MATLAB. Prior to the measurement, Gaussian filter (20 pixels) is applied on the images to decrease the outlier pixels and noise in the raw image. In the code, the binarized images of segmented patches are first rotated so that the line connecting the center of the prism and one of the prism tips aligns with x-axis. The tip of the prism is assigned as $x = 0$. Note that $w(x)$ at $x < 0$ region was measured by subtracting the prism width $p(x)$ from the width of patch contour at x. The 10 representative $w(x)$ profiles are averaged along the x-axis and standard deviation was obtained for each x.

The tip-to-tip distance d and interparticle angle γ between nanoprism pair were measured as following. The “oval” function (in circle mode) in ImageJ is used to fit six rounded tips from two nanoprisms in one nanoprism pair connected by the merged patches. The center coordinates and radii of the fitted circles are the inputs into MATLAB to compute the interparticle angle γ between the nanoprism pair using our customized codes. We first connect the center of one prism, which is defined as the mean of three centers of the circles fitted from each tip, with the center of the circle fitted from the tip with the merged polymer patch in the same nanoprism, to draw a nanoprism orientation vector. The angle between two lines from two nanoprisms in a pair (annotated in Fig. 4a scheme and Fig. 4d y-axis ticks) is then calculated and plotted as the interparticle angle γ as shown in Fig. 4d. The tip-to-tip distance d is measured as the center-to-center distance between two circles fitted from two tips connected by the merged polymer patches in a nanoprism pair subtracted by the sum of two radii.

Supplementary Note 6: Scaling behavior for patch size and shape

The patch area A can be described as $A \sim \delta RD$, where δ is the fractional coverage of polymer chains over the adsorption sites and D is the corresponding interchain separation. Fractional coverage δ exhibits a grafting temperature dependence $\delta \sim T^{-9/2}$. This scaling exponent stems from a combination of the temperature dependence of adsorption on a flat surface $(-3/2)$ and the effect of surface curvature (-3). From our scaling theory, $R \sim \alpha^{1/5}T^{-1}$ and $D \sim \alpha^{-1/2}$. T^{-1} reflects the Arrhenius behavior of χ. Scaling exponents for α of $1/5$ and $-1/2$ reflect good solvent and standard interchain polymer brush spacing scaling, respectively. Combining all terms results in a scaling of $A \sim \alpha^{-3/10}T^{-11/2}$.

In addition to the crossover interaction parameter χ^*, we additionally employ Eq. 11 – 23 to predict the shape of the patches that form from symmetry breaking grafting. Due to differences in chain-chain and chain-solvent interactions, we can leverage traditional wetting relationships to predict the surface curvatures of the resulting patchy grafts as a function of distance from the prism core’s surface r. Our governing balance can be derived as follows. Starting from the Young-Laplace wetting relationships we have

$$\gamma_{\mathrm{SG}} = \gamma_{\mathrm{SL}} + \gamma_{\mathrm{LG}} \cos \theta_{\mathrm{c}} \tag{28}$$

where θ_c is the contact angle and γ_{SL}, γ_{LG}, and γ_{SG} are the surface tension costs of core–chain, chain–solvent, and core–solvent contacts, respectively. γ_{SG} is taken to be negligible since particles are freely

dispersed in solution. Rearranging gives $\cos\theta_c = \gamma_{SL}\gamma_{LG}^{-1}$. The surface tensions can be expressed in terms of energy per unit area $\gamma_{SL} = \varepsilon_{SL}A_c^{-1}$ and $\gamma_{LG} = \varepsilon_{LG}A_c^{-1}$, where A_c is the contact area. Plugging in gives

$$\cos\theta_c \sim [\varepsilon_{SL}/\varepsilon_{LG}] \quad (29)$$

where as we assume that the contact area A_c are of the same order of magnitude (within a constant scaling constant). Therefore, the contact angle can be approximated as the ratio of the energy gain of chain–chain/core contact versus the energy cost of chain–solvent contacts for a given layer at distance from the prism core surface r. $\varepsilon_{SL}(r)$ and $\varepsilon_{LG}(r)$ can be readily computed using the surface area SA and volume V of a conical frustum at every radial layer at r from the surface.

$$SA = \pi(R_1 + R_2)\sqrt{(R_1 - R_2)^2 + h^2} \quad (30)$$

$$V = \frac{1}{3}h(R_1^2 + R_1R_2 + R_2^2) \quad (31)$$

where h is the height of the frustrum and R_1 and R_2 are the radii of the larger and smaller circular faces, respectively. Here, our physical picture is of increasingly bigger correlation blobs ξ with larger radial distance from the core's surface. Thus, both radii and h are given by the size of the blob, giving

$$SA(r) = \pi\left[\xi(r) + \xi\big(r + \xi(r)\big)\right]\sqrt{\left[\xi(r) - \xi\big(r + \xi(r)\big)\right]^2 + h^2} \quad (32)$$

$$V(r) = \frac{1}{3}h\left[\xi^2(r) + \xi(r)\xi\big(r + \xi(r)\big) + \xi^2\big(r + \xi(r)\big)\right] \quad (33)$$

The relevant energies are then $\varepsilon_{SL}(r) \sim V(r)\psi(r)$ and $\varepsilon_{LG}(r) \sim SA(r)\psi^{2/3}(r)$. Using Eq. 32 – 33, we can then solve for the contact angle θ_c as a function of distance from the nanoparticle's surface and construct the patch shape as shown in Fig. 3f.

Supplementary Note 7: Grafting density estimation from TEM images

We calculate the grafting density of polymers on each patch[13] based on patch width $w(x)$ and tip of the prism width $p(x)$ in Supplementary Note 5, to investigate the patch shape-dependent grafting density. First, the volume of grafted polymers per patch V_{patch} at each reaction condition was obtained based on TEM images of the patches following Eq. 34, assuming that $\frac{1}{2}\big(w(x) + p(x)\big)$ and $\frac{1}{2}p(x)$ are circularly symmetric around the axis connecting the tip and center of the patches, based on the TEM tomographs of the tip area. The calculated V_{patch} for each condition are summarized in Supplementary Table 5.

$$V_{\text{patch}} = \int \pi\left(\frac{1}{2}\big(w(x) + p(x)\big)\right)^2 - \pi\left(\frac{1}{2}p(x)\right)^2 dx \quad (34)$$

The molar volume of polymer chain V_{pspaa} is estimated to be 4.15×10^{25} nm^3/mol from Eq. 35 by plugging in the molar weight of PS block $M_{\text{n(ps)}}$ and PAA block $M_{\text{n(paa)}}$ as 1.6×10^4 g/mol and 3.7×10^4 g/mol, respectively, and the density of PS ρ_{ps} and PAA as ρ_{paa} as 1.05×10^{-21} g/nm^3 and 1.41×10^{-21}

g/nm^3, respectively.

$$V_{\mathrm{pspaa}} = \left(\frac{M_{\mathrm{n(ps)}}}{\rho_{\mathrm{ps}}}\right) + \left(\frac{M_{\mathrm{n(paa)}}}{\rho_{\mathrm{paa}}}\right) \tag{35}$$

Then the number of polymer chain per patch n_{chain} is calculated following Eq. 36, where the Avogadro's number N_{A} is 6.023×10^{23} mol^{-1}. The n_{chain} obtained from each condition is shown in the Supplementary Table 5.

$$n_{\mathrm{chain}} = \left(\frac{V_{\mathrm{patch}}}{V_{\mathrm{pspaa}}}\right) \times N_{\mathrm{A}} \tag{36}$$

Next, the surface area of nanoprisms covered by a patch A_{patch} are calculated for each reaction condition using Eq. 37 – 38 and average patch chord length l in Fig. 3d, average prism thickness t_{prism} of 27.2 nm, and the average prism tip radius r_{tip} of 5.5 nm as detailed in Supplementary Fig. 1. In detail, A_{patch} can be approximated as a combination of three geometric shapes: two equal triangles a_1, four trapezoids a_2, and a one third of the surface area of a prolate spheroid S_{prolate} a_3 as illustrated in Supplementary Fig. 15.

$$A_{\mathrm{patch}} = 2a_1 + 4a_2 + a_3 \tag{37}$$

$$\begin{aligned} A_{\mathrm{patch}} = {} & 2 \times \frac{\sqrt{3}}{4}\left(l - \frac{4}{\sqrt{3}} r_{\mathrm{tip}}\right)^2 \\ & + 4 \times \sqrt{r_{\mathrm{tip}}{}^2 + (\frac{1}{2} t_{\mathrm{prism}})^2} \times \frac{1}{2}\left(l - \sqrt{3} r_{\mathrm{tip}} + l - \frac{4}{\sqrt{3}} r_{\mathrm{tip}}\right) \\ & + \frac{1}{3} S_{\mathrm{prolate}} \end{aligned} \tag{38}$$

where the surface area of the prolate spheroid S_{prolate} is 783.3 nm^2 from the Eq. 39, the semi-minor axis is the prism tip radius r_{tip}, a semi-major axis is the half of the prism thickness t_{prism}, and the eccentricity z is defined as Eq. 40.

$$S_{\mathrm{prolate}} = 2\pi r_{\mathrm{tip}}{}^2 \left(1 + \frac{t_{\mathrm{prism}}}{2r_{\mathrm{tip}} \cdot z} \arcsin z\right) \tag{39}$$

$$z^2 = 1 - \left(\frac{2r_{\mathrm{tip}}}{t_{\mathrm{prism}}}\right)^2 \tag{40}$$

Last, the grafting density σ' is obtained as dividing the number of polymer chains per patch n_{chain} by the surface area covered by the patch A_{patch} as Eq. 41.

$$\sigma' = n_{\text{chain}}/A_{\text{patch}} \tag{41}$$

For example, at reaction condition of T = 90 °C and α = 25 nM, the volume of the patch V_{patch} is 11862.9 ± 5725.0 nm^3 by using the Eq. 34 and the average chord length is 16.8 nm. Therefore, the number of polymer chains n_{chain} is 172.2 ± 83.1 by the Eq. 36. The surface area of nanoprism covered by the patch A_{patch} is 606.0 nm^2 from the Eq. 38. Thus the grafting density σ' of the single-patch prisms obtained is 0.28 ± 0.14 nm^{-2}. Similarly, A_{patch} and σ' are obtained for the patches from different reaction conditions and summarized in Supplementary Table 5.

Supplementary Note 8: Patch-patch merging and binary assembly scaling

Here, we posit that patch–patch merging is facilitated by free ends of chains contacting with those from another grafted prism. As such, merging can only occur at a sufficient local chain concentration c^*. This is readily given using the overlap concentration crossover $c^* \sim NfR^{-3}$

$$c^* \sim r_{\text{o}}^{4/5}\sigma^{2/5}v_{\text{o}}^{-3/5}[1-2\chi]^{-3/5}b_l^{-3}N^{-4/5}\Omega^{9/5} \tag{42}$$

For concentrations greater than c^*, grafted chains enter the semi-dilute regime. Here, grafted chains are split into two regions – outer and inner. The inner region near prism core is dominated by chain-chain interactions between grafted on the same core. Conversely, the outer region is influenced by chains from other grafted particles. The crossover between the two regimes can be defined by where the monomer concentration $\psi(r)$ is equal to the bulk concentration in semi-dilute regime $b_l^3 c$, giving $\psi(R_L) \sim b_l^3 c$. Solving for the crossover radial distance R_{L} gives

$$R_{\text{L}} \sim r_{\text{o}}\sigma^{1/2}v_{\text{o}}^{-1/4}[1-2\chi]^{-1/4}b_l\Omega^{-3/2}\left[b_l^3 c\right]^{-3/4} \tag{43}$$

Using Eq. 43, we can determine the number of monomers N_{c} in R_{L} equating it to Eq. 23

$$R_{\text{L}} \sim R(N_c) \sim r_{\text{o}}\sigma^{1/5}v_{\text{o}}^{1/5}[1-2\chi]^{1/5}b_l^{2/5}\left[\frac{N_c}{\Omega}\frac{b_l}{r_{\text{o}}}\right]^{3/5} \tag{44}$$

Solving for N_{c} gives

$$N_{\text{c}} \sim r_{\text{o}}\sigma^{1/2}v^{-3/4}[1-2\chi]^{-3}\Omega^{-3/2}\left[b_l^3 c\right]^{-5/4} \tag{45}$$

The correlation size of outer regime chain R_{x} is a function of the local concentration c and can be readily computed using classical semi-dilute scaling relation: $R_{\text{x}} \sim N_{\text{x}}^{1/2}b_l\left\{b_l^3 c[v_o(1-2\chi)]^{-1}\right\}^{-1/8}$, where the number of monomers in R_{x} is $N_{\text{x}} \sim N - N_{\text{c}}$. Therefore, the chain size in the limit at semi-dilute region R_{sd} where patches are interacting and overlapping with each other is $R_{\text{sd}} \sim R_{\text{L}} + R_{\text{x}}$, giving Eq. 46.

$$R_{\mathrm{sd}} \sim r_{\mathrm{o}}\sigma^{1/2}b_l\Omega^{-3/2}[\nu_{\mathrm{o}}(1-2\chi)]^{-1/4}\left(b_l^3c\right)^{-3/4} + (N-N_{\mathrm{c}})^{1/2}b\left[\frac{b_l^3c}{\nu_{\mathrm{o}}(1-2\chi)}\right]^{-1/8} \tag{46}$$

Taking the ratio of Eq. 47 to Eq. 23 allows us to predict interpenetration ratio λ between two interacting polymer patches shown in Fig. 4f as following: $\lambda \sim 1 - R_{\mathrm{sd}}/R$.

Note that in terms of the PAA conformation between the tips, we model the merging PAA blocks as a spherical packing of correlation blobs to fill up the space available between the tips. This picture is a manifestation of the idea that introducing charge moieties results in a reorganization of the charged PAA monomers into neutral correlation blobs that then interact via steric repulsion with each other. As discussed above (Supplementary Note 8), this assumption enables the usage of a classical semi-dilute scaling relationship, $N_{\mathrm{x}}^{1/2}b_l\left\{b_l^3c[\nu_o(1-2\chi)]^{-1}\right\}^{-1/8}$, between sterically interacting correlation blobs to model both the correlation size as well as the effective width of the merging regime between the two interacting tips.

Supplementary Note 9: Localized surface plasmon resonance (LSPR) properties of the patch-to-patch assembled prisms

We calculated LSPR properties of four types of patch merging driven assemblies of symmetry breaking patchy nanoprisms, namely the nano-bowties, quasi-linear and branched trimers, and tetramer as shown in Fig. 4a, Supplementary Figs. 17 and 18. The extinction spectra (Supplementary Figs. 17q , 18y) and electric field distribution maps for the corresponding structures show that nanoprisms have configuration-dependent extinction energies and “hot spots”, where the electric fields are a few orders of magnitude more intense than the surroundings. The nano-bowties (Supplementary Fig. 17a,e,i,m) present the face, side and tip modes[14,15] upon excitation with visible light. The dimer in Supplementary Fig. 17a, for example, exhibit the face, edge, and tip modes at 2.81 2.72, and 2.33 eV respectively (Supplementary Fig. 17b–d). As the particles are brought closer together with larger γ (Supplementary Fig. 17i,m), the respective modes redshift and the electric field intensity is shown to spread in between the space of the assembled particles, visualizing a stronger interaction between particles. The hot spots visualized in the electric field maps possess a relative field intensity enhancement close to ~ 8×10^7, compared to the near vanishing intensity ~200 nm away from the nanoprisms. The corresponding optical extinction spectra of structures in air, to mimic the dried-state assembly are compared to the spectrum of a single-patch nanoprism before merging (‘single’ in Fig. Supplementary Fig. 17q).

Likewise, the quasi-linear trimer (Supplementary Fig. 18a) has the LSPR peaks at energies corresponding to 2.81, 2.69, 2.31 and 2.27 eV (Supplementary Fig. 18b–e), with “hot spots” showing up over the face, edge, and tip of each nanoprism. The dipolar tip mode further splits into the antisymmetric antibonding and symmetric bonding modes present at higher and lower energies, respectively. As the particles are brought closer (Supplementary Fig. 18f), the edge and tip modes show an appreciable interaction with the electric field intensity being confined in the space between the nanoprisms (Supplementary Fig. 18i,j), with a redshift in the antibonding mode from 2.31 eV to 2.29 eV compared to the structure in Supplementary Fig. 17a. We further calculate the electromagnetic enhancements of branched trimer and the tetramer shown in Fig. 4a (Supplementary Fig. 18o,s, respectively). The branched trimer (Supplementary Fig. 18o) has one particle pointing up, resulted in the shift of face modes from 2.81 to 2.85 eV, while edge mode remains unchanged at 2.69 eV, and the tip mode blue-shifted from the nondegenerate energies at 2.31 and 2.27 eV to 2.29 and 2.25 eV, respectively. The tetramer (Supplementary Fig. 18s) exhibits hot spots around the truncated particle present near the top of the assembly, in addition to the usual face, edge and tip modes. The extinction peak at 2.47, 2.29, and 2.27 eV (Supplementary Fig.

18v,w,x, respectively) shows the electric field localized at the non-truncated face of the particle, the tips of non-truncated particles, with some delocalization occurring in between these particles. The LSPR energy shifts of various structures are represented in the extinction spectra obtained from the structures in air (Supplementary Fig. 18y), with the spectrum corresponding to the trimer shown in Fig. 4g.

Supplementary Tables 1–6

Supplementary Table 1. Reaction conditions for synthesizing patchy nanoprisms with varied patch count per prism n and shapes as shown in Fig.2d,f, and 3a. The reaction procedures and definition of stock solution II are detailed in Methods. The α is the ratio of 2-NAT molar concentration to the optical density of the prism suspension at its maximum extinction wavelength.

α (nM)	T (°C)	Volume of stock solution II (μL)	Volume of water (μL)	Volume of DMF (μL)	Volume of 2-NAT solution (2 mg/mL in DMF, μL)	Volume of PS-*b*-PAA solution (8 mg/mL in DMF, μL)
50	90	100	100	818	2	80
50	100	100	100	818	2	80
50	110	100	100	818	2	80
25	90	100	100	819	1	80
37.5	90	100	100	818.5	1.5	80
75	90	100	100	817	3	80

Supplementary Table 2. Fractions of patchy nanoprisms with varied patch count n obtained at different temperature T at fixed α of 50 nM as shown in Fig. 2e. The "Other" refers to prisms with at least one prism edge fully coated.

T (°C)	Zero-patch	Single-patch	Double-patch	Triple-patch	Other	Patch count per prism n	Standard deviation of n	Number of particles analyzed
90	0	0.136	0.673	0.164	0.028	1.97	0.55	214
100	0	0.105	0.250	0.640	0.006	2.52	0.69	172
110	0	0	0.010	0.985	0.005	2.98	0.10	200

Supplementary Table 3. Fractions of patchy nanoprisms with varied patch count n obtained at different α and fixed temperature T of 90 °C as shown in Fig. 2g. The "Other" refers to prisms with at least one prism edge fully coated.

α (nM)	Zero-patch	Single-patch	Double-patch	Triple-patch	Other	Patch count per prism n	Standard deviation of n	Number of particles analyzed
25	0.091	0.818	0.085	0	0.006	0.99	0.42	330
37.5	0.009	0.462	0.417	0.085	0.027	1.55	0.65	223
75	0	0.051	0.328	0.610	0.011	2.54	0.59	177

Supplementary Table 4. Reaction conditions for synthesizing 2-NAT coated prisms for Raman characterizations as shown in Supplementary Fig. 8 (see Methods for more details). Note that we did not add PS-*b*-PAA in the reaction. Still, the total volume ratio of DMF to water in each reactor vial was fixed to be 4.5 to 1, consistent with the conditions for single-patch prism synthesis.

α (nM)	*T* (°C)	Volume of stock solution II (μL)	Volume of water	Volume of DMF (μL)	Volume of 2-NAT solution (2 mg/mL in DMF) (μL)	Volume of PS-*b*-PAA solution (8 mg/mL in DMF)
25	90	200	-	898	2	-
50	90	200	-	896	4	-
75	90	200	-	894	6	-
50	100	200	-	896	4	-
50	110	200	-	896	4	-

Supplementary Table 5. Average grafting density σ' per patch obtained from reaction conditions in Fig. 3a (Calculation detailed in Supplementary Note 7). Note that standard deviation of the each grafting density come from the volume of the patch V_{patch}.

α (nM)	T (°C)	Volume of the patch V_{patch} (nm^3)	Number of chains per patch n_{chain}	Chord length l (nm)	Surface area covered by the patch A_{patch} (nm^2)	Grafting density σ' (nm^{-2})
25	90	11862.9 ± 5725.0	172.2 ± 83.1	16.8	606.0	0.28 ± 0.14
50	90	8647.3 ± 2327.1	125.5 ± 33.8	18.4	713.2	0.18 ± 0.05
75	90	9071.5 ± 4627.2	131.6 ± 67.2	19.2	768.5	0.17 ± 0.09
50	100	4125.1 ± 3402.2	59.9 ± 49.4	16.3	573.4	0.10 ± 0.09
50	110	1936.5 ± 497.5	28.1 ± 7.2	16.1	560.5	0.05 ± 0.01

Supplementary Table 6. Control experiment of single-patch prisms self-assembly via simple charge screening (see Methods for the details).

Volume of single-patch nanoprism solution (μL)	Volume of 200 mM NaCl (μL)	Final concentration of NaCl (mM)
9	1	22.2
8	2	40
4	3	85.7

Supplementary Figures 1–21

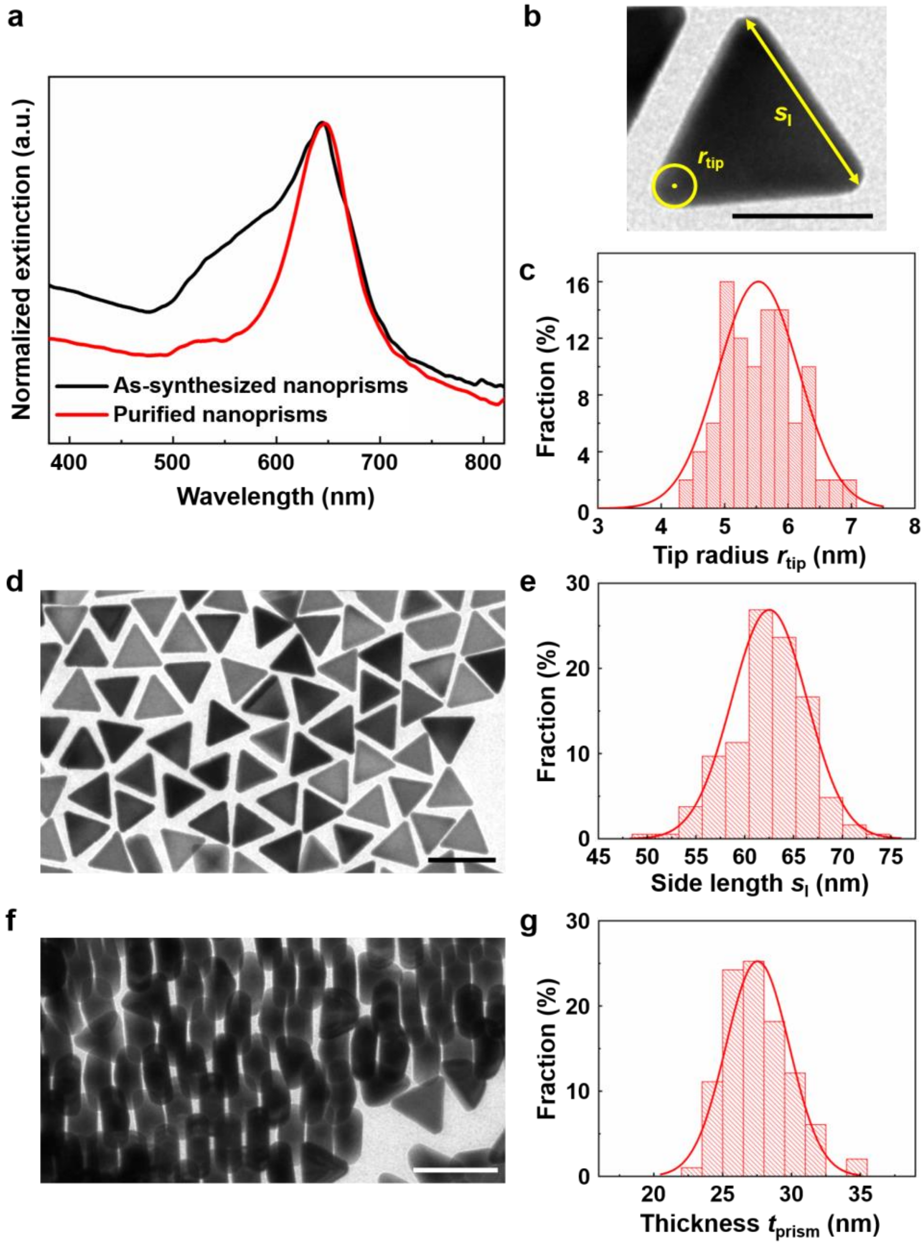

Supplementary Fig. 1. Purification and dimensions of gold triangular nanoprisms (a) Normalized UV-Vis spectra of the as-synthesized gold prisms before purification (black), and after purification, directly used for the patch formation reactions (red). (b) Representative high magnification TEM image of nanoprism noted with edge length s_{l} and tip radius r_{tip}. (d,f) TEM images of prisms after purification (d) top-view and (f) side view. (c,e,g) Histogram of the (c) prism tip radius r_{tip}, (e) edge length s_{l}, and (g) prism thickness t_{prism} (r_{tip} = 5.5 ± 0.6 nm, s_{l} = 62.5 ± 3.9 nm, t_{prism} = 27.2 ± 3.7 nm). 70–100 nanoprisms are measured for each parameter. Scale bars: 50 nm (b). 100 nm (d,f).

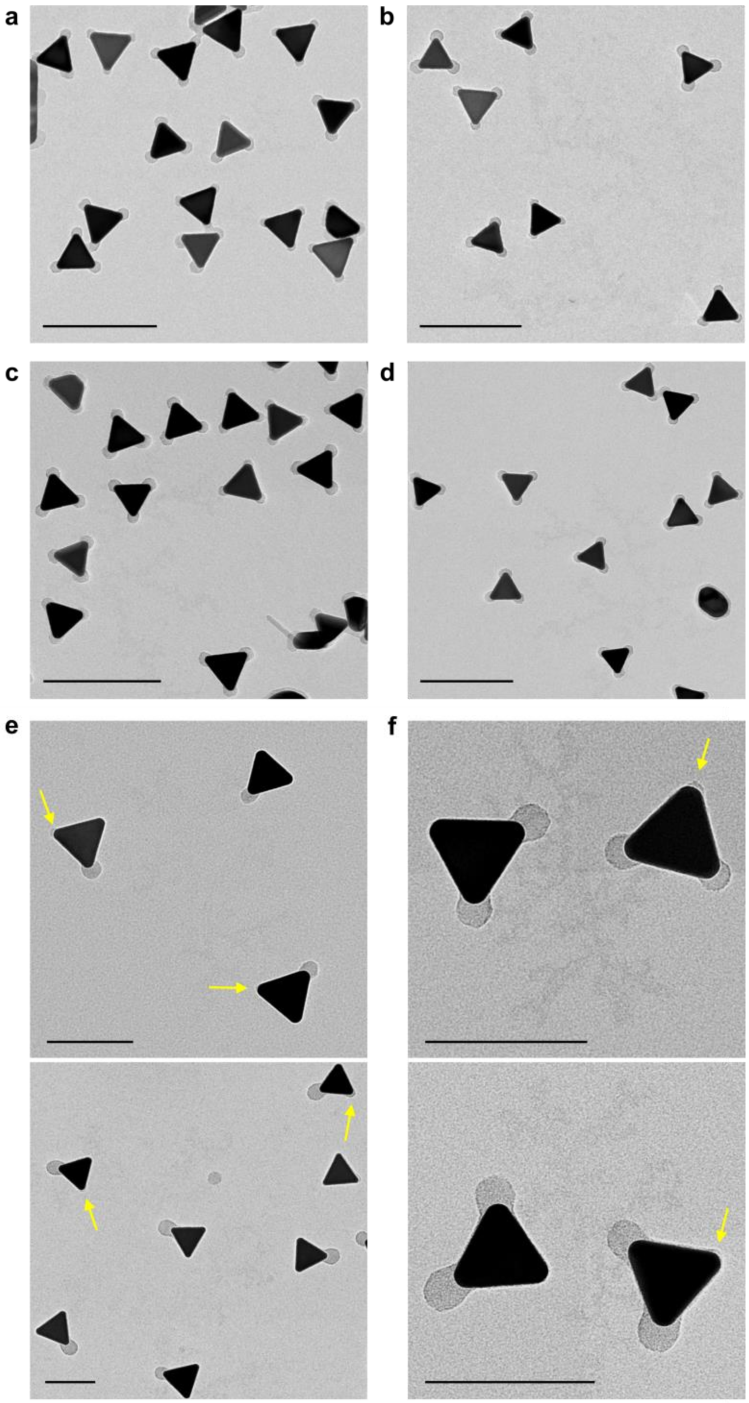

Supplementary Fig. 2. 2-naphthalenethiol (2-NAT) adsorption on all three tips of nanoprism. (a–d) Triple-patch prism formation while α is varied as following: (a) 25 nM, (b) 37.5 nM, (c) 50 nM, (d) 75 nM at fixed temperature of 110 °C and polymer concentration of 30 nM. We can deduce that the range of 2-NAT concentrations used in our experiments can lead to the similar amount of thiol adsorption on all three nanoprism tips. Furthermore, the amount of polymers used in each reaction is also sufficient to cover all three prism tips.[11] (e,f) Trace amount of thin polymer layers on the tips other than the tips occupied by patches (highlighted by yellow arrows), implying polymers can adsorb on all three tips of prisms. Grafting temperature T is fixed at 90 °C while α is varied as following: (e) 25 nM, (f) 50 nM. Scale bars: 200 nm (a–d), 100 nm (e,f).

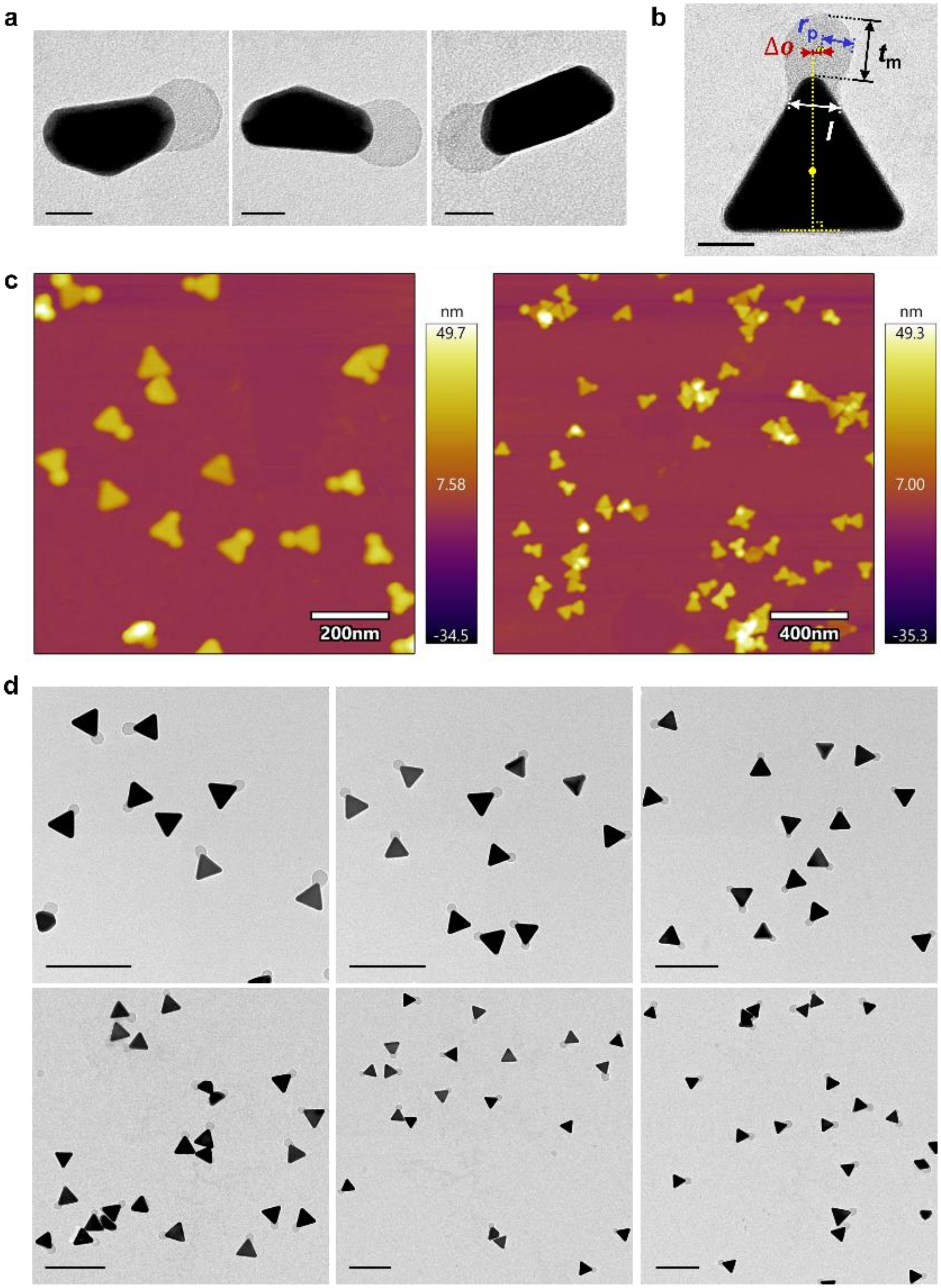

Supplementary Fig. 3. Single-patch prisms dimensions. (a) TEM images of single-patch prims from the side view. (b) A TEM image of single-patch nanoprism noted with four geometric variables. Presented are the chord length l, the maximum patch thickness t_{m}, the fitted-inscribed circle radius r_{p} of the patch contour, the offset of the patch center Δo defined as the distance from centroid of the fitted inscribed circle of the patch contour to the line connecting the prism center and the tip center. (c) AFM images of single-patch nanoprisms. (d) Low magnification TEM images of single-patch prisms. Scale bars: 20 nm (a,b), as noted in the image (c), and 200 nm (d).

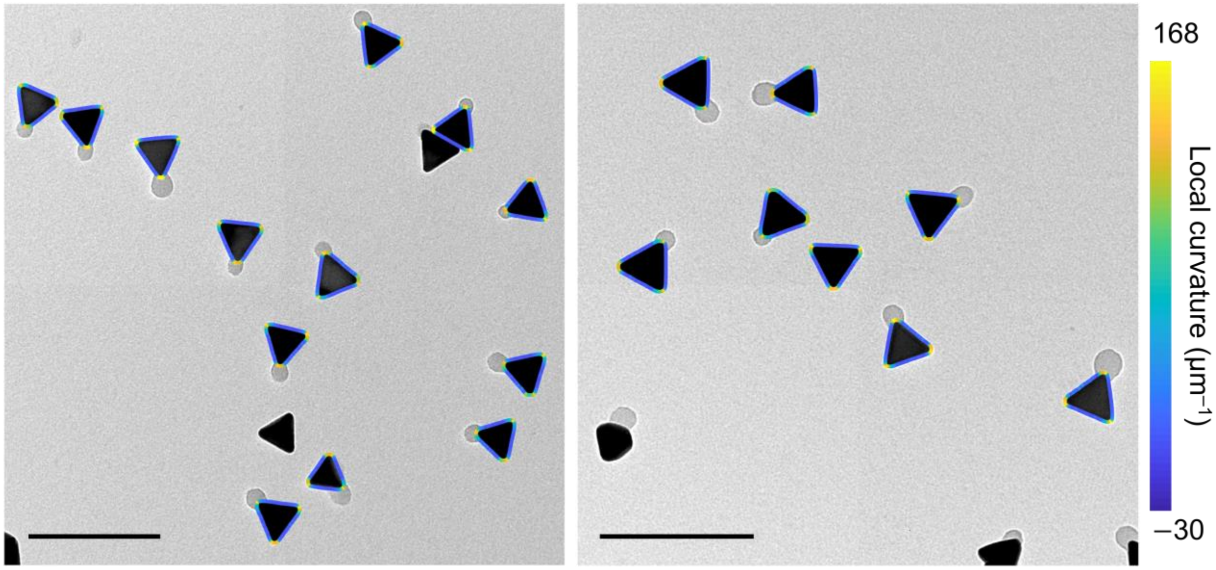

Supplementary Fig. 4. Local curvature analysis of prism contours. The curvatures of nanoprism tips are similar for all three tips. The patches are not necessarily formed on the tip of the highest curvature among three, differentiating presented polymer–polymer attraction-induced asymmetric grafting from the previously reported curvature induced patch formation[1,7,16]. Scale bars: 200 nm.

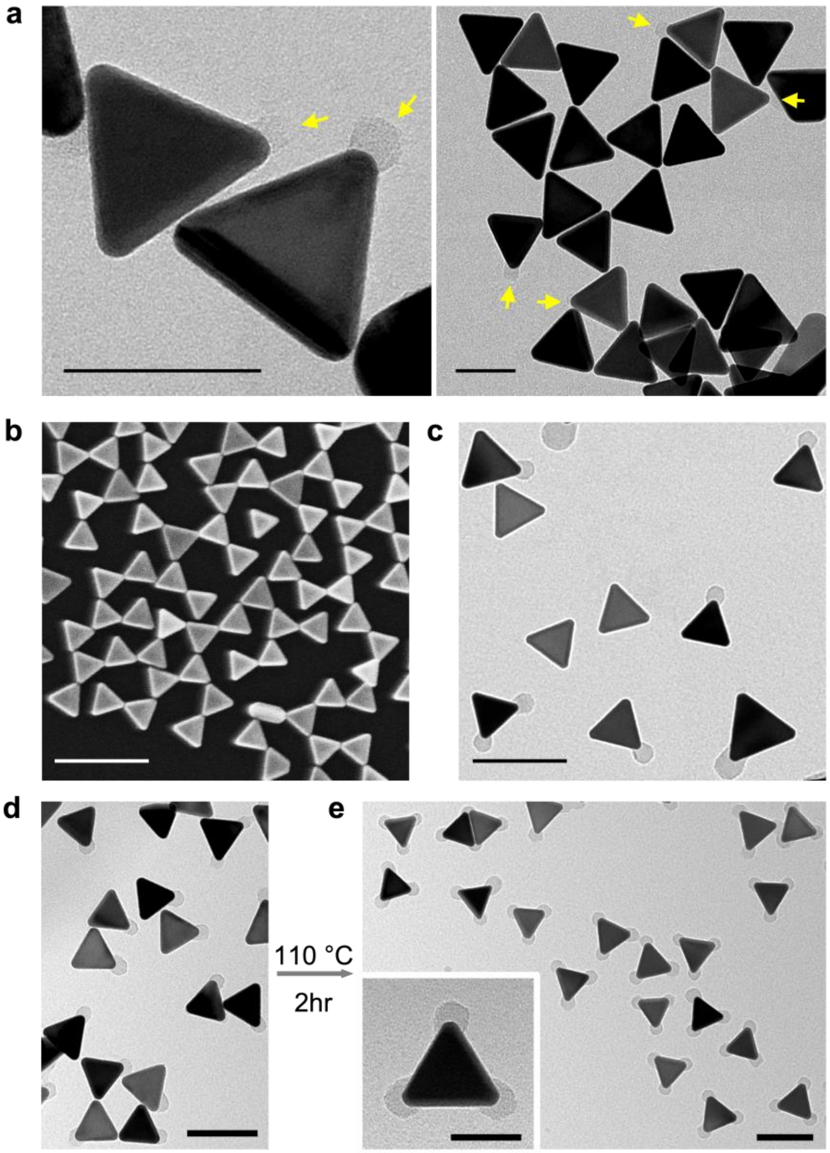

Supplementary Fig. 5. 2-NAT decorates all three tips and asymmetric grafting happens from the early stage of the reaction. (a) Representative TEM images after 10% of the total reaction time at $\alpha = 50$ nM and $T = 90$ °C, followed by quick quenching (Methods). Patches are highlighted by the yellow arrows. (t_m = 5.8 ± 2.3 nm, l = 8.7 ± 1.0 nm). This intermediate reaction time allows fewer polymers to be grafted, producing a more flattened patch shape compared to longer reaction times (100% of the total reaction time), while preserving the single-patch geometry. (b) SEM image of nanoprisms decorated with 2-NAT-only, without polymers (α = 25 nM, same as that in the single-patch prism synthesis condition in Fig. 1, see Methods), assembling by their tips due to hydrophobic attraction between 2-NAT covered tips. (c) TEM image obtained from sequential addition of PS-*b*-PAA into 2-NAT-only coated prisms. Note that polymer patches are formed asymmetrically, despite that all three tips are coated by 2-NAT. (d) TEM image of single-patch prisms prepared at T = 90 °C and α = 25 nM. (e) TEM image of tri-patch prisms obtained by incubating single-patch prisms in (d) at 110 °C for another 2 h. This result shows that asymmetric patch grafting is reversible. Scale bars: 50 nm (a), 200 nm (b), 100 nm (c), 100 nm (d,e), 50 nm (inset in e).

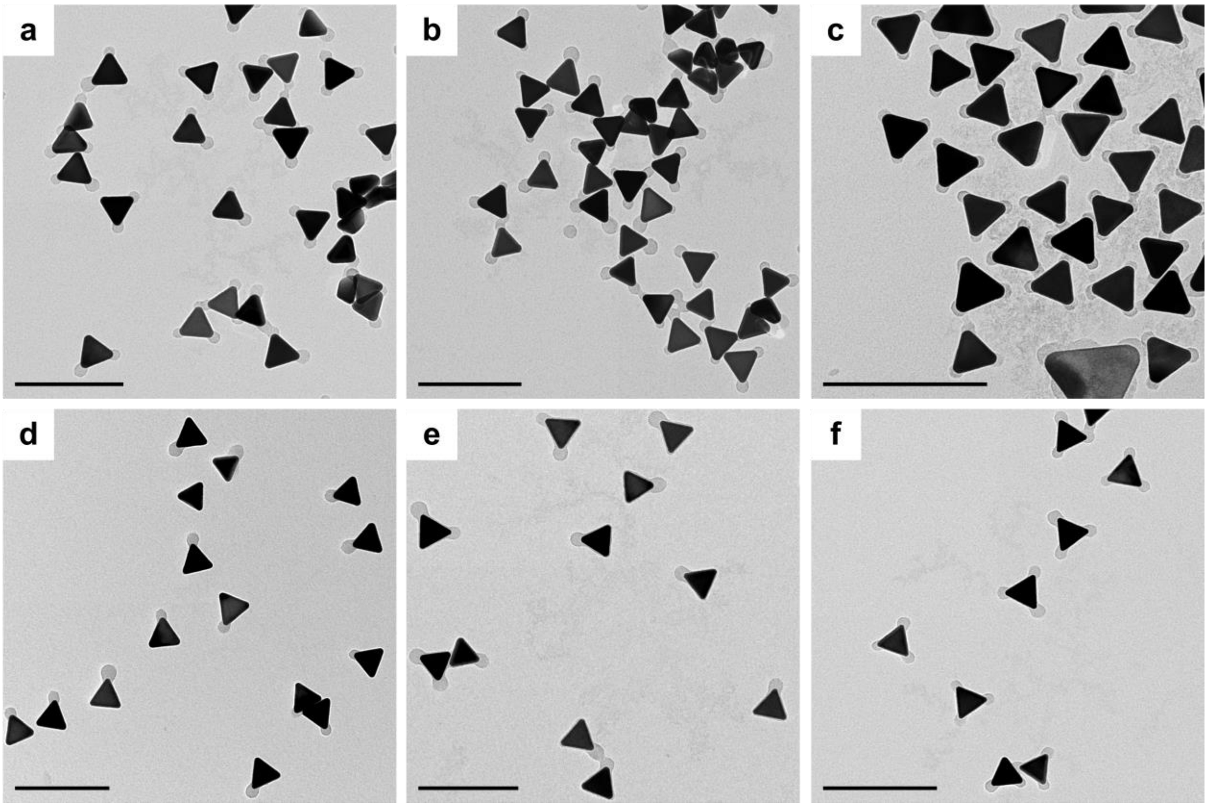

Supplementary Fig. 6. Low magnification TEM images of patchy nanoprisms obtained at different conditions. (a–c) T and (d–f) α variation. (a) 90 °C, (b) 100 °C, (c) 110 °C at a fixed α of 50 nM. (d) 25 nM, (e) 37.5 nM, (f) 75 nM at a fixed T of 90 °C. All conditions have fixed polymer concentration of 30 nM. Scale bars: 200 nm.

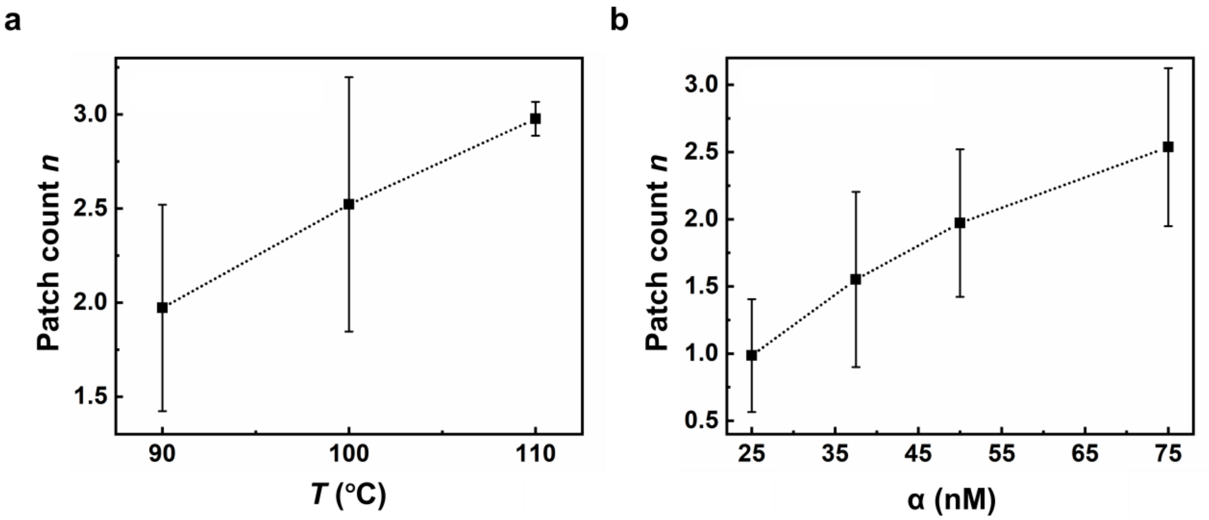

Supplementary Fig. 7. Average patch count per prism obtained from varied reaction conditions. Average patch count per prism during the change of (a) grafting temperature T from 90 to 110 °C at fixed α of 50 nM and (b) α from 25 to 75 nM at a fixed T of 90 °C. The error bars are from the standard deviation.

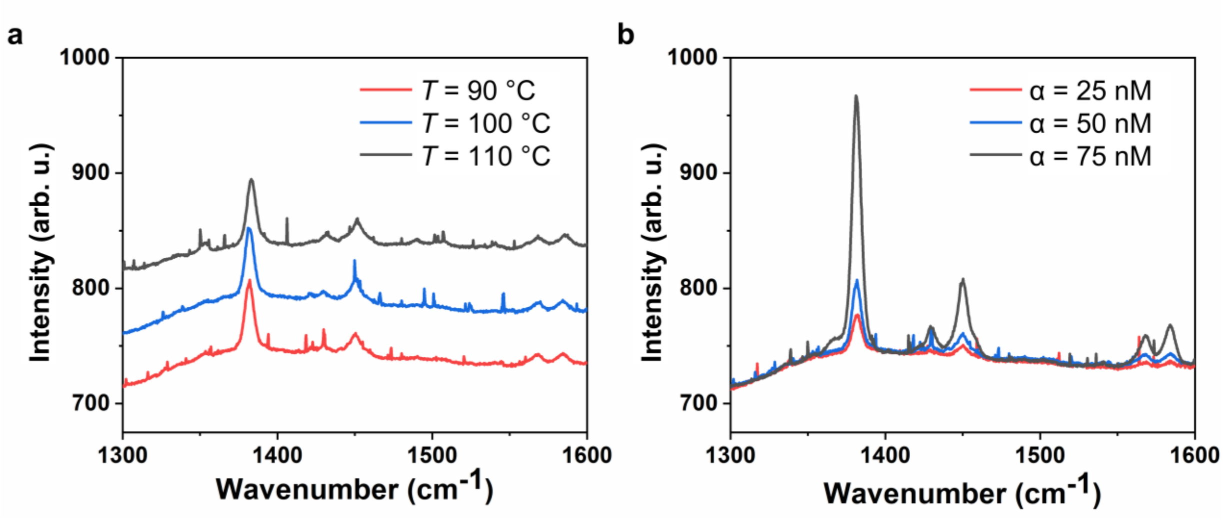

Supplementary Fig. 8. Raman spectra of 2-NAT coated gold nanoprisms. (a,b) Raman spectra of 2-NAT coated gold nanoprisms obtained by changing (a) grafting temperature T from 90 to 110 °C at a fixed α of 50 nM and (b) α from 25 to 75 nM at fixed T of 90 °C. (a) T = 90 °C (red), 100 °C (blue), and 110 °C (black). (b) α = 25 nM (red), 50 nM (blue), and 75 nM (black). The peaks at 1374, 1423, and 1444 cm^{-1} correspond to the stretching modes of polycyclic aromatic hydrocarbon ring in 2-NAT.[17,18]

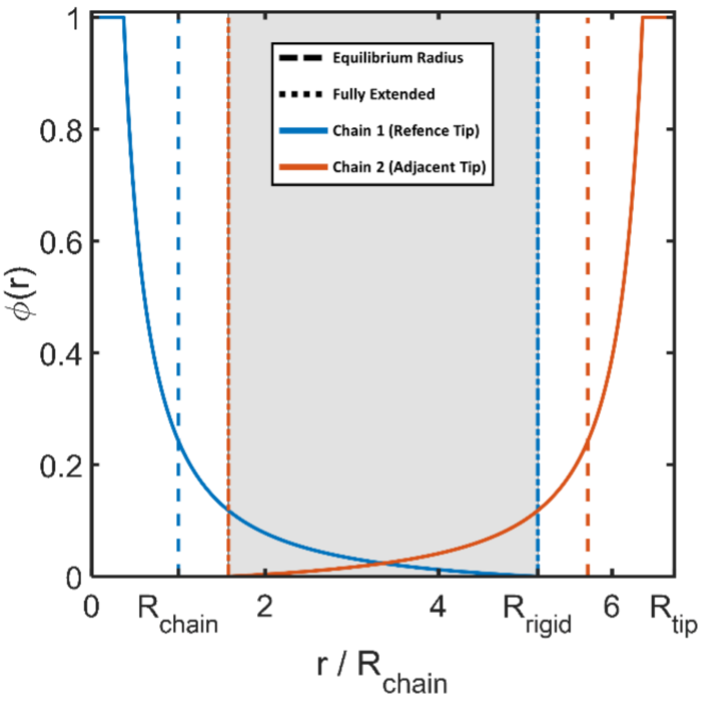

Supplementary Fig. 9. Monomer density distribution $\phi(r)$ prediction as a function of distance from the tip. As detailed in Supplementary Note 4, one chain is placed at a reference tip (r = 0) and the other at a distance equal to the edge length of the prism (R_{tip}). Single-dashed lines mark the equilibrium, hydrodynamic size of the tip-decorating chain (R_{chain}) and double-dashed lines indicate the fully extended size of the chain (R_{rigid}). Colors correspond to the same chain (blue: chain at reference tip, orange: chain at adjacent tip) and the x-axis is scaled by R_{chain}. The gray shading region indicates the space where the chains' monomer densities overlap with each other. This means that any monomer within this region will experience strong monomer–monomer interactions that ultimately drive chain reorganization and subsequent merging to a single tip.

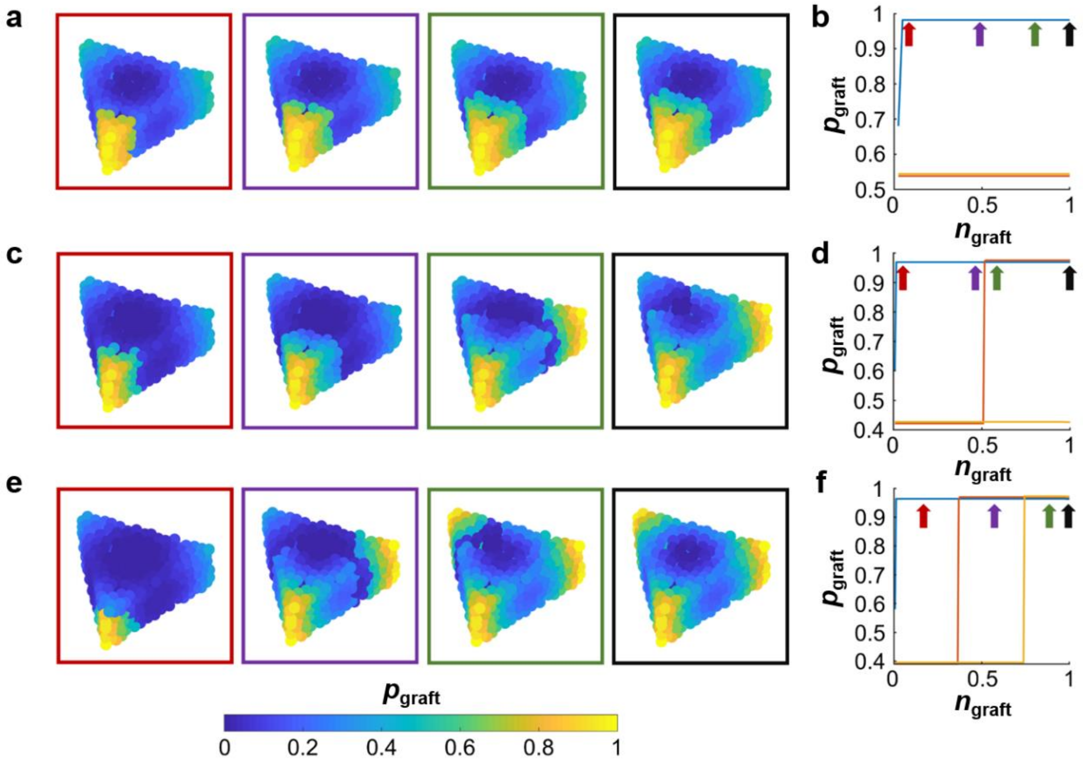

Supplementary Fig. 10. Evolution of local grafting probability at varied α. Snapshots from MC simulation showing evolution of local grafting probability of polymers p_{graft} upon α increase from the top to the bottom row (a,c,e) and corresponding graph highlighting p_{graft} on the tips (colored blue, red, yellow) as a function of fraction of grafted chains n_{graft} (b,d,f). Colored arrows on p_{graft} profiles correspond to simulation snapshots at each n_{graft} boxed in same colors (a,c,e).

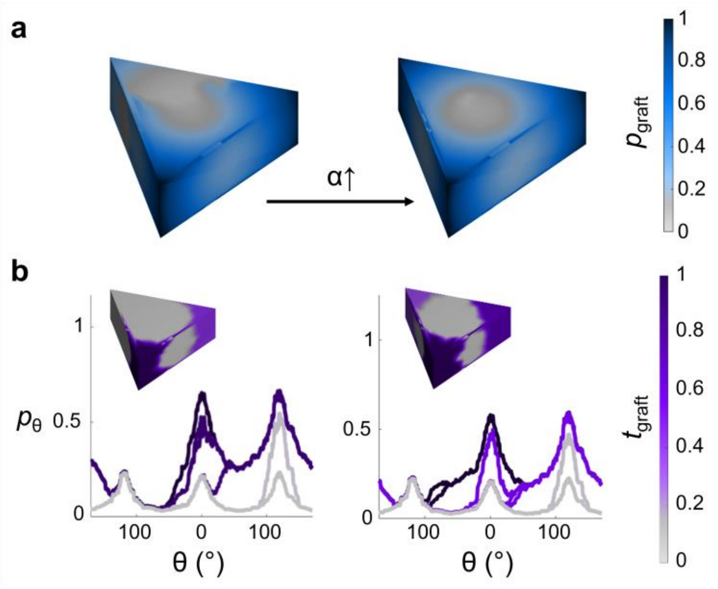

Supplementary Fig. 11. Grafting probability at varied *α*. (a) Local grafting probability of polymers p_{graft} upon α increase. (b) Grating probability of an incoming chain p_θ as a function of angle θ over the prism contour (labeled in Fig. 2a) colored by the extent of grafting simulation time t_{graft}.

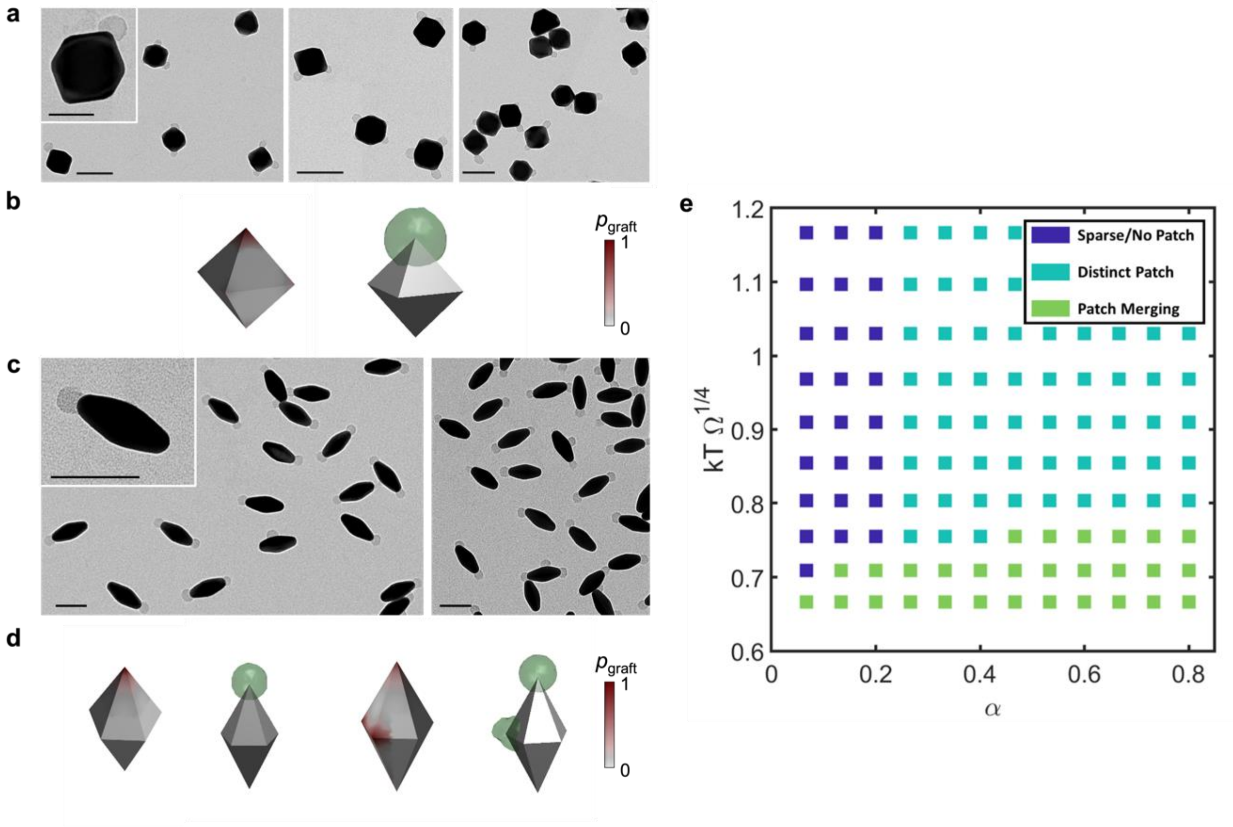

Supplementary Fig. 12. Extension of asymmetric grafting to other NP shapes. (a,c) Asymmetric grafting on (a) gold octahedra and (c) gold bipyramids obtained at 90 °C. (b,d) 3D grafting probability p_{graft} and the representative simulated morphology of (b) patchy octahedron and (d) patchy bipyramids corresponding to (a) and (c), respectively. (e) Phase diagram of patch morphologies for octahedra and bipyramid. Ω here is a proxy for shapes to enable collapse of both sets of shape data into one phase diagram. Scale bars: 50 nm (a), 100 nm (c).

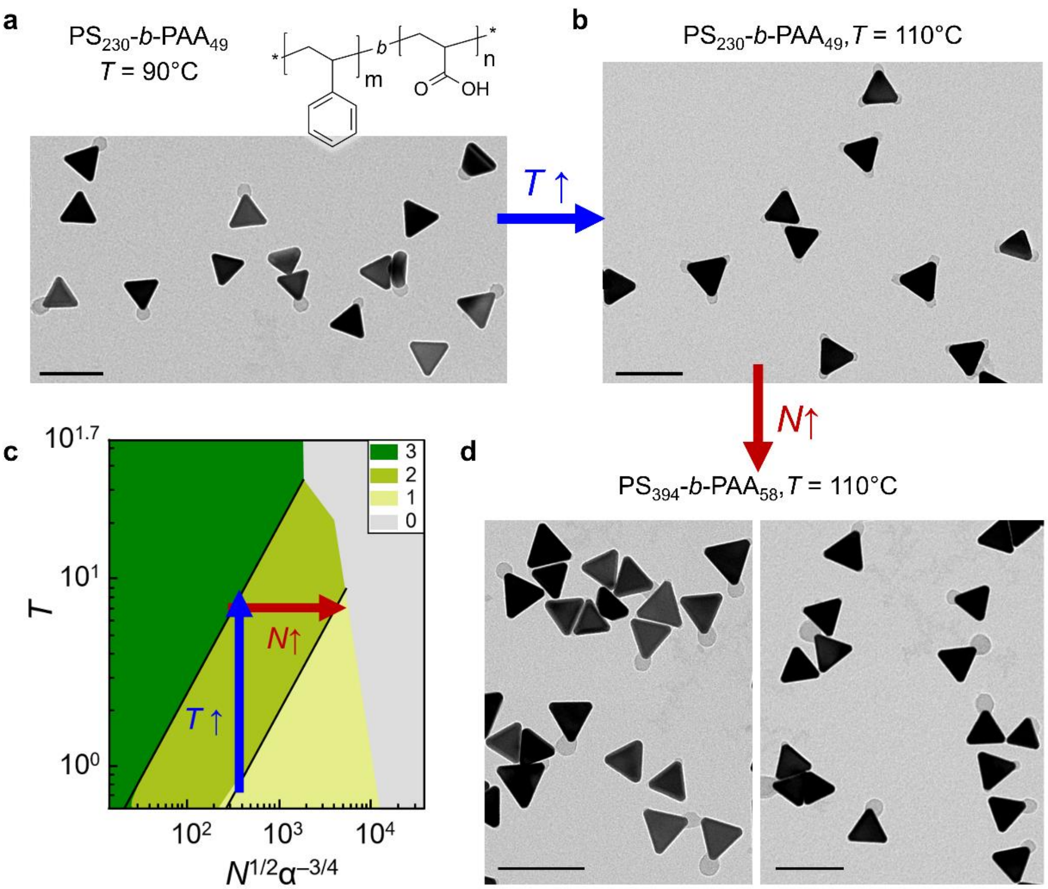

Supplementary Fig. 13. Extension of asymmetric grafting to other chain length. (a,b) TEM images of single- and tri-patch nanoprisms obtained by using PS_{230}-*b*-PAA_{49} at T of (a) 90 °C and (b) 110 °C, at a fixed α of 25 nM. (c) Phase diagram of patch count n (shading colored to n as noted in the legend) across the parameter space: grafting temperature T, chain length N, and ligand concentration α. The phase diagram is partitioned to different colored regions following simulation results. The black lines are phase boundaries predicted by theory. Arrows show illustrations of estimated corresponding experimental variation in (a,b,d). (d) TEM images of single-patch nanoprisms obtained using PS_{394}-*b*-PAA_{58} at T = 110°C and α = 25 nM. Scale bars: 100 nm.

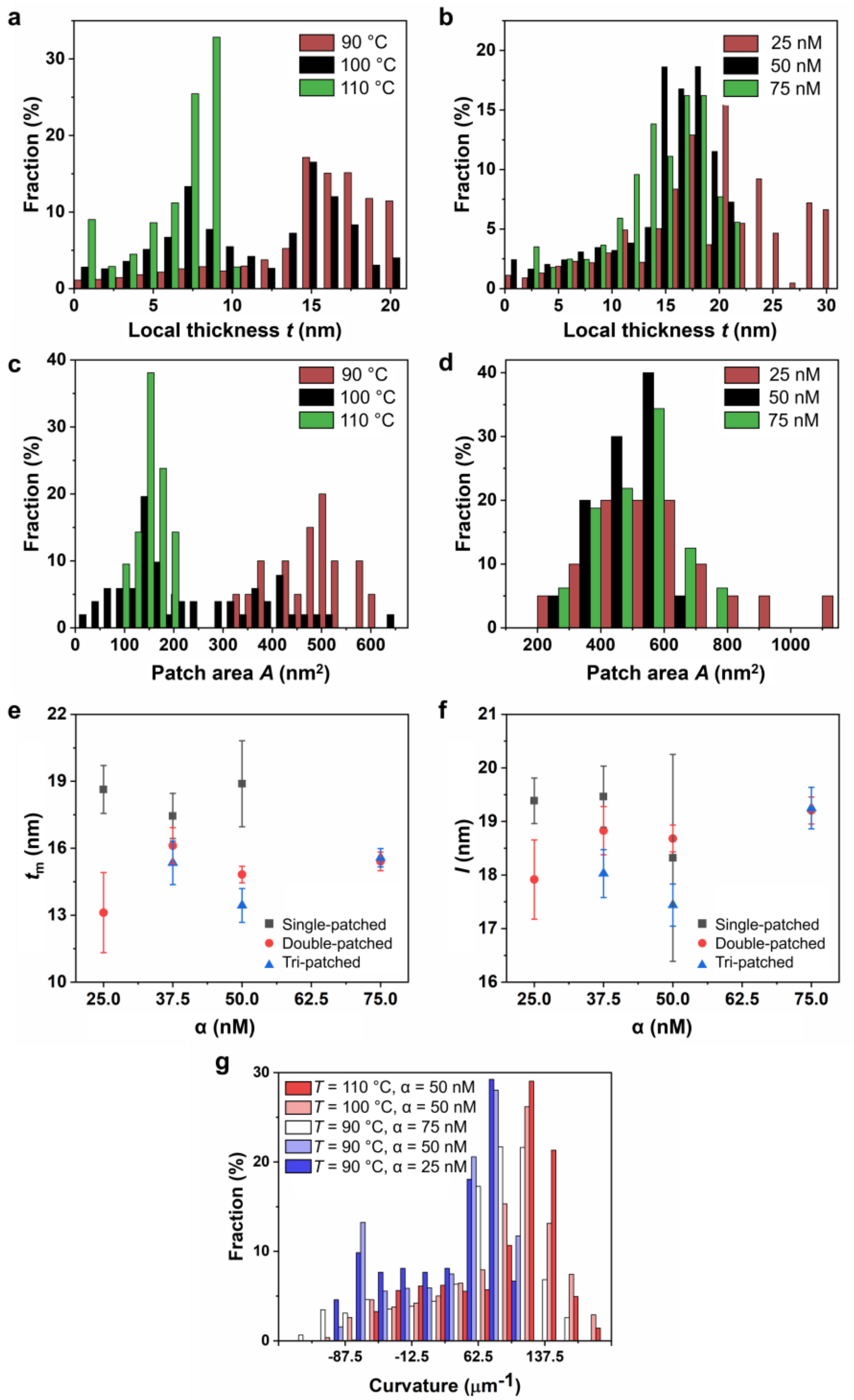

Supplementary Fig. 14. Dimension and shape analysis of the patches. (a,b) Histogram of area local thickness of patches obtained from conditions in Fig. 3a at (a) varied grafting temperature T with fixed the ratio of 2-NAT to NP concentration to OD of the prisms α of 50 nM and (b) varied α at the fixed T of 90 °C. (c,d) Histogram of patch area A obtained from conditions in Fig. 3c at (c) varied T with fixed α of 50 nM and (d) varied α at the fixed T of 90 °C. (e,f) Statistics of (e) patch thickness t_m and (f) chord length l for each patch count obtained at varied α at fixed T of 90 °C. Note that t_m and l of single-patch prism are slightly larger, in general, compared to those of the double- and tri-patch prisms. The error bars are from the standard deviations. (g) Histogram of patch contour local curvature obtained from varied reaction conditions as in Fig. 3e.

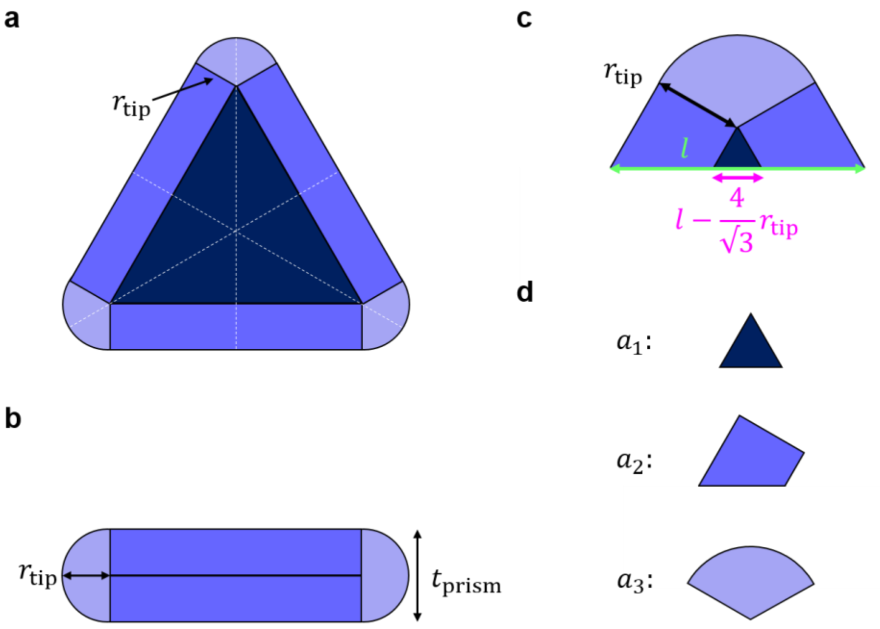

Supplementary Fig. 15. Approximation of nanoprism tip surface area covered by a patch. (a,b) Geometric approximation of a nanoprism from (a) the top and (b) the side views, which can be described by the combination of equal triangles, rectangles, and one third of prolate. (c) Zoom-in view of a nanoprism tip of radius r_{tip} covered by a patch with the chord length l measured from 2D projected TEM view. (d) The surface area of prism tip covered by a patch can be estimated by the three composing shapes in (c): equal triangle a_1 with edge length of $l-\frac{4}{\sqrt{3}}r_{\mathrm{tip}}$, trapezoid a_2 with edge lengths of $l-\frac{4}{\sqrt{3}}r_{\mathrm{tip}}$ and $l-\sqrt{3}r_{\mathrm{tip}}$ with height of $\sqrt{r_{\mathrm{tip}}{}^2+(\frac{1}{2}t_{\mathrm{prism}})^2}$, and a one third of prolate surface area a_3 with semi-major and semi-minor axis of t_{prism} and r_{tip}, respectively (Calculation details in Supplementary Note 7 and Table 5).

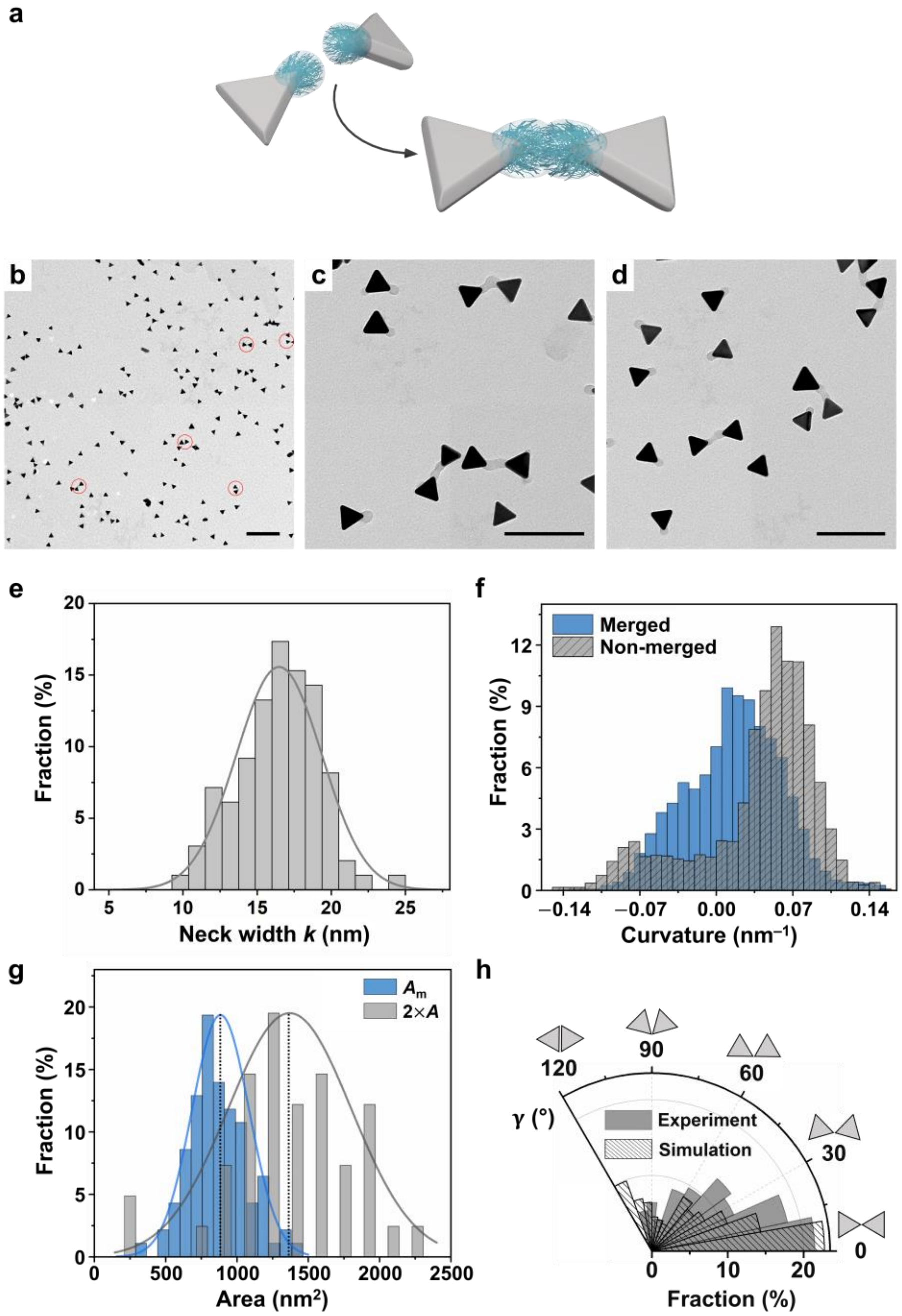

Supplementary Fig. 16. Patch-to-patch merging into bowties. (a) Scheme of tip-to-tip dimerization via patch merging. (b–d) Low mag images of single-patch prisms assembled into dimers via patch-patch merging. Dimers assembled by patch- patch merging are highlighted with red circles in (b). Patch-to-patch merging density in (b): 0.27 pair/μm^2. (e) Neck width k at the merged interface (93 pairs are measured). (f) Curvature of the non-merged and merged patches in comparison (42 single-patch prisms and 93 pairs are compared). (g) Projected patch area of merged patches A_m decreases by 35.4% compared to the two times of single-patch prisms' patch area A, based on the following equation: $1 - A_\mathrm{m}/2A$. (A_m = 887 ± 200 nm^2. 41 single-patch prisms and 45 patch-merged pairs are measured). (h) Interparticle angular distribution γ from experiment (solid bar) and simulation (striped bars). Scale bars: 500 nm (b), 200 nm (c,d).

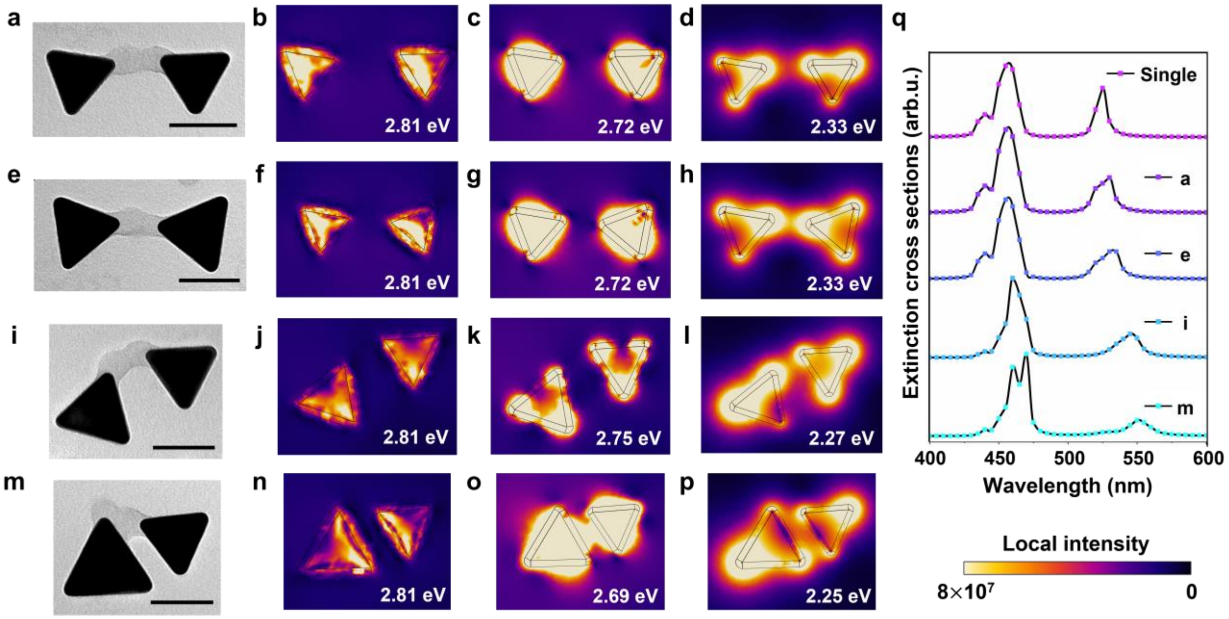

Supplementary Fig. 17. Spatial electric field distribution maps of bowties. (a,e,i,m) The electron microscope images of the gold nanoprisms assembled into dimer configuration of different angles. The spatial electric field distribution maps for each configuration correspond to near field enhancements at extinction maxima are represented using false color maps on the same row (b–d, e–h, i–l, m–p). The excitation energies are noted in the maps. (q) The respective optical extinction spectra of structures in air compared with the spectrum corresponds to the single-patch prism before merging (noted as “single prism”). Scale bars: 50 nm (a,e,i,m).

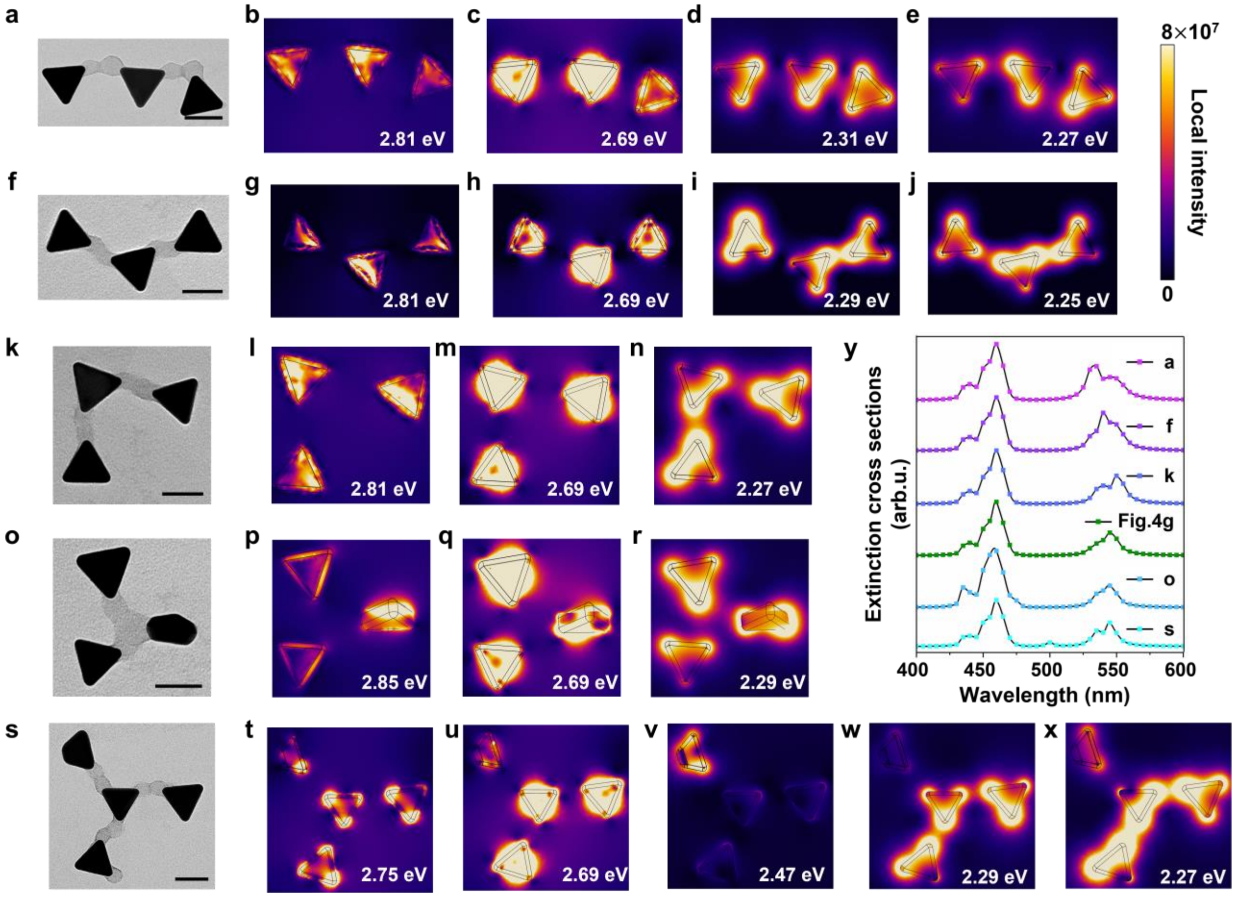

Supplementary Fig. 18. Spatial electric field distribution maps of trimers and tetramers. (a,f,k,o,s) TEM images of the gold nanoprisms assembled into trimers with (a,f) quasi-linear, (k) angled, and (o) branched configuration, and (s) tetramer. The spatial electric field distribution maps for each configuration correspond to near field enhancements at extinction maxima are represented using false color maps on the same row (b–e, g–j, i–n, l–n, p–r, t–x). The excitation energies are noted in the maps. (y) The respective optical extinction spectra of the structures in air compared with the spectrum corresponds to the trimer in Fig. 4g. Scale bars: 50 nm (a,f,k,o,s)

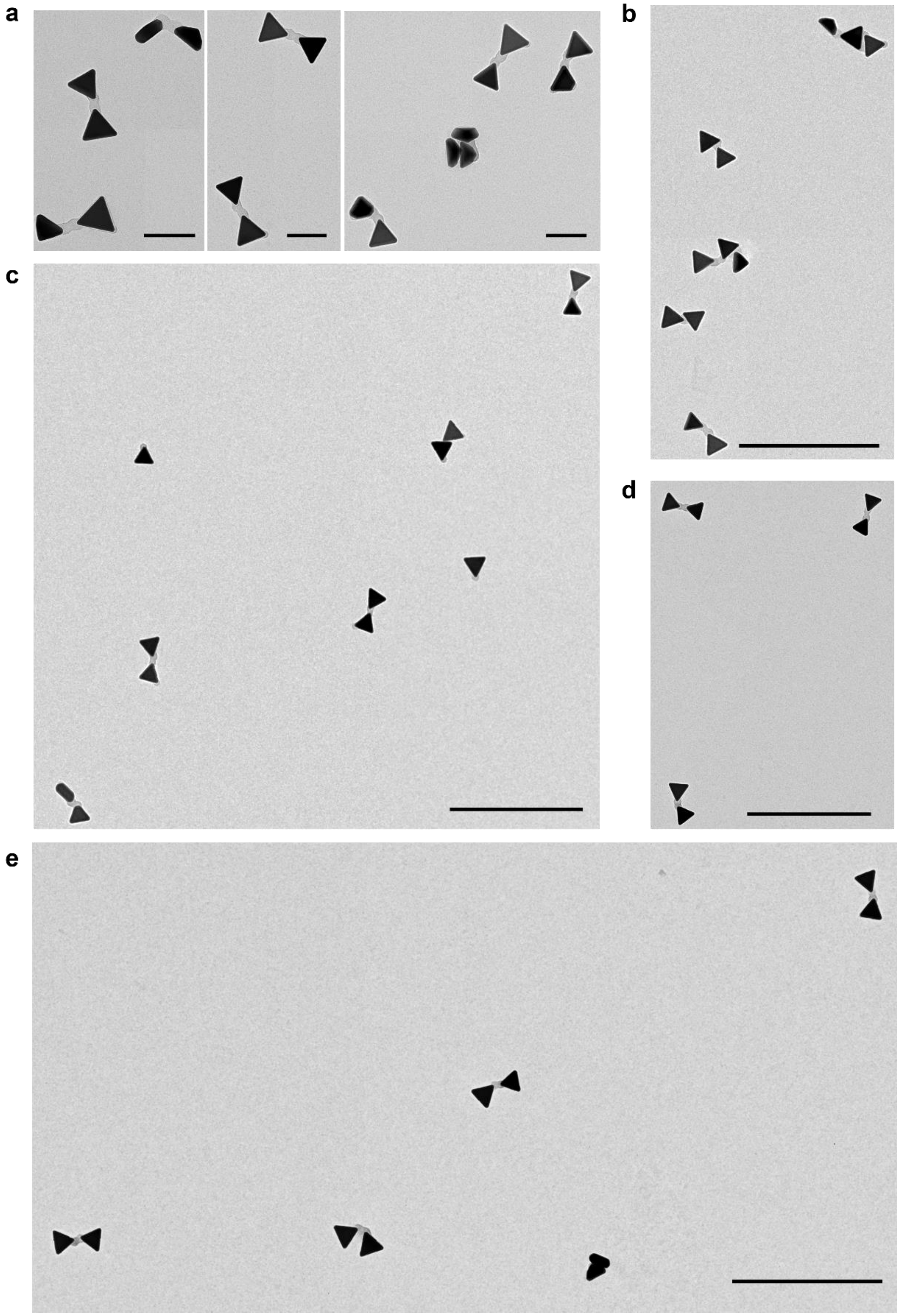

Supplementary Fig. 19. TEM images of single-patch prisms assembled into dimers via CTAB-induced patch-patch selective complexation. Scale bars: 100 nm (a), 500 nm (b–e).

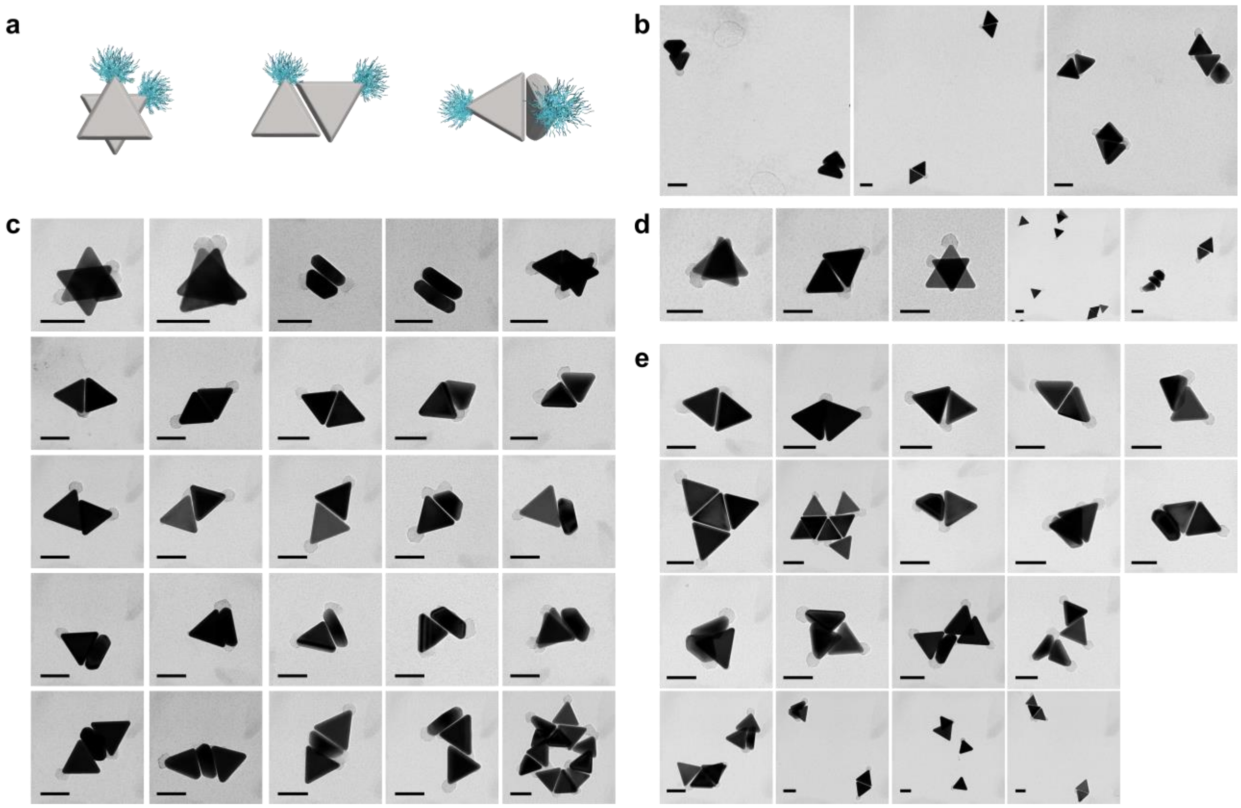

Supplementary Fig. 20. NaCl induced assembly of single-patch prisms. (a) Schematics of the dimeric assembly motifs, whose assembly are governed by van der Waals force between gold prism surfaces. (b,c) Low magnification TEM images (b) and high magnification TEM images (c) of NaCl induced assembly at 22.2mM. (d,e) TEM images of NaCl induced assembly at 40 mM (d) and 85.7 mM (e). Scale bars: 50 nm.

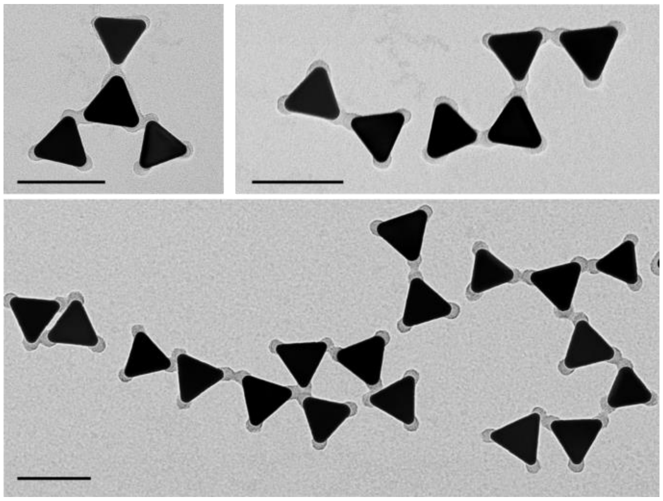

Supplementary Fig. 21. TEM images of tri-patch prisms assembled into networks via patch-patch selective assembly. Scale bars: 100 nm.

Supplementary References

1. Elbert, K. C. *et al.* Dendrimer Ligand Directed Nanoplate Assembly. *ACS Nano* **13**, 14241–14251 (2019).
2. Lu, F. *et al.* Unusual packing of soft-shelled nanocubes. *Sci. Adv.* **5**, eaaw2399 (2022).
3. Bates, F. S. & Fredrickson, G. H. Block Copolymer Thermodynamics: Theory and Experiment. *Annu. Rev. Phys. Chem.* **41**, 525–557 (1990).
4. Elbert, K. C. *et al.* Anisotropic nanocrystal shape and ligand design for co-assembly. *Sci. Adv.* **7**, eabf9402 (2022).
5. Borukhov, I. & Leibler, L. Enthalpic Stabilization of Brush-Coated Particles in a Polymer Melt. *Macromolecules* **35**, 5171–5182 (2002).
6. Aubouy, M. & Raphaël, E. Surface-tethered polymers in polymeric matrices. *J. Phys. II* **3**, 443–448 (1993).
7. Lu, F. *et al.* Unusual packing of soft-shelled nanocubes. *Sci. Adv.* **5**, eaaw2399.
8. Elbert, K. C. *et al.* Anisotropic nanocrystal shape and ligand design for co-assembly. *Sci. Adv.* **7**, eabf9402.
9. Mai, Y. & Eisenberg, A. Self-assembly of block copolymers. *Chem. Soc. Rev.* **41**, 5969–5985 (2012).
10. Liu, C. *et al.* Toroidal Micelles of Polystyrene-block-Poly(acrylic acid). *Small* **7**, 2721–2726 (2011).
11. Kim, A. *et al.* Tip-Patched Nanoprisms from Formation of Ligand Islands. *J. Am. Chem. Soc.* **141**, 11796–11800 (2019).
12. Kim, J. *et al.* Reconfigurable Polymer Shells on Shape-Anisotropic Gold Nanoparticle Cores. *Macromol. Rapid Commun.* **39**, 1800101 (2018).
13. Duan, H., Luo, Q., Wei, Z., Lin, Y. & He, J. Symmetry-Broken Patches on Gold Nanoparticles through Deficient Ligand Exchange. *ACS Macro Lett.* **10**, 786–790 (2021).
14. Borodinov, N. *et al.* Enhancing hyperspectral EELS analysis of complex plasmonic nanostructures with pan-sharpening. *J. Chem. Phys.* **154**, 014202 (2021).
15. Agrawal, A. *et al.* Resonant Coupling between Molecular Vibrations and Localized Surface Plasmon Resonance of Faceted Metal Oxide Nanocrystals. *Nano Lett.* **17**, 2611–2620 (2017).
16. Nie, Z. *et al.* Self-assembly of metal–polymer analogues of amphiphilic triblock copolymers. *Nat. Mater.* **6**, 609–614 (2007).
17. Agarwal, N. R. *et al.* SERS detection and DFT calculation of 2-naphthalene thiol adsorbed on Ag and Au probes. *Sens. Actuators B Chem.* **237**, 545–555 (2016).
18. Alvarez-Puebla, R. A., Jr, D. S. D. S. & Aroca, R. F. Surface-enhanced Raman scattering for ultrasensitive chemical analysis of 1 and 2-naphthalenethiols. *Analyst* **129**, 1251–1256 (2004).
