# Supplementary material for: Symmetry-breaking in patch formation on triangular gold nanoparticles by asymmetric polymer grafting
Source: Nat Commun. 2022 Nov 9;13:6774. doi: 10.1038/s41467-022-34246-0 (PMC9646788; doi:10.1038/s41467-022-34246-0)
Supplement: Supplementary file 3 — Description of Additional Supplementary Files [file 41467_2022_34246_MOESM3_ESM.pdf]

Supplementary Movie 1: Animated 3D rendering and slices of single-patch nanoprisms from TEM tomography.

Supplementary Movie 2: Visualization of asymmetric grafting effect driving changes in chain grafting probability $p$graft from simulation.

Supplementary Movie 3: Animated 3D rendering and slices of dimeric prisms with merged patches from TEM tomography.
